# Chemical tuning of quantum spin–electric coupling in molecular magnets

**DOI:** 10.1038/s41557-025-01926-5

**Published:** 2025-08-27

**Authors:** Mikhail V. Vaganov, Nicolas Suaud, François Lambert, Benjamin Cahier, Christian Herrero, Régis Guillot, Anne-Laure Barra, Nathalie Guihéry, Talal Mallah, Arzhang Ardavan, Junjie Liu

**Affiliations:** 1 CAESR, Department of Physics, University of Oxford, The Clarendon Laboratory, Oxford, UK; 2 Laboratoire de Chimie et Physique Quantiques (LCPQ), Université de Toulouse, CNRS, Toulouse, France; 3 Institut de Chimie Moléculaire et des Matériaux d’Orsay, Université Paris-Saclay, CNRS, Orsay, France; 4 Laboratoire National des Champs Magnétiques Intenses, CNRS, Université Grenoble Alpes, Grenoble, France; 5 School of Physical and Chemical Sciences, Queen Mary University of London, London, UK

**Keywords:** Chemical physics, Magnetic materials, Computational chemistry

## Abstract

Controlling quantum spins using electric rather than magnetic fields promises substantial architectural advantages for developing quantum technologies. In this context, spins in molecular magnets offer tunability of spin–electric couplings (SECs) by rational chemical design. Here we demonstrate systematic control of SECs in a family of Mn(II)-containing molecules by varying the coordination environment of the spin centre. The trigonal bipyramidal (tbp) molecular structure with *C*_3_ symmetry leads to a substantial molecular electric dipole moment that is directly connected to its magnetic anisotropy. The interplay between these two features gives rise to experimentally observed SECs, which can be rationalized by wavefunction theoretical calculations. Our findings guide strategies for the development of electrically controllable molecular spin qubits for quantum technologies.

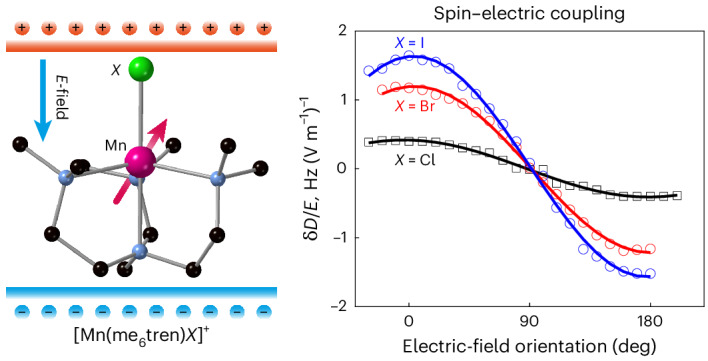

## Abstract (web summary)

Molecular magnets may serve as engineerable spin qubit candidates for quantum information science; however, the magnetic fields often used for control can be challenging to confine. Now, it has been shown that well-designed mononuclear Mn(II) complexes demonstrate enhanced spin–electric coupling, providing guidance for electrically controllable molecular spin qubits.

## Main

The possibility of electrical spin control offers substantial architectural advantages for classical or quantum spintronics because, compared to magnetic fields, electric fields can be efficiently routed and confined in complex nanoscale circuits, thereby reducing energy consumption and facilitating logic operations on spins^1–5^. Research into interactions between electric fields and spin degrees of freedom in various quantum systems have attracted interest both theoretically and experimentally^6–12^. A strong spin–electric coupling (SEC) is critical both for efficient electrical quantum spin control and for engineering coherent spin–electric interfaces that allow the exchange of quantum information between distinct spin qubits^13,14^.

Among the candidates for spin qubits, molecular magnets offer particular advantages: coordination chemistry allows rich tunability of molecular quantum spin structures while also providing routes to large-scale integration via supramolecular approaches^15–18^. One approach to enhancing SECs in molecular magnets^19–22^ is to exploit strong spin–orbit coupling (SOC) by employing heavy metals, such as rare earth atoms, as the spin carrier. For example, a Ho(III)-containing molecular magnet, in which a small structural distortion from strict tetragonal symmetry leads to an *E*-field-sensitive spin transition, demonstrates a SEC that is sufficiently strong to enable selective spin control using modest electric fields of 10^5^ V m^−1^ (ref. ^22^). Although providing important insights, the Ho(III) example also highlights a limitation of this approach: such molecules typically possess a giant ligand field-induced magnetic anisotropy, leading to inconveniently large transition energies between spin states. Furthermore, the origin of the SEC in this system is an accidental symmetry-breaking, rather than the result of rational chemical design. We thus identify a challenge of engineering molecular magnets with spin transitions that are both in an accessible energy range and sensitive to electric fields.

The *S* = 5/2 spin associated with a Mn(II) ion is a simple quantum system with potential for quantum information processing (QIP). As a free ion, it has a half-filled 3*d* shell with an electron ground state of *S* = 5/2 and *L* = 0. In molecular or crystalline environments, the weak SOC leads to small magnetic anisotropies, reduced spin–lattice relaxation and impressive spin relaxation times. It also removes one of the key ingredients for enhancing SEC, at first sight compromising the scope for efficient *E*-field spin control^23–25^. Indeed, so far, a sizable SEC in Mn(II) has only been observed when doped into ferro- or piezoelectric hosts^26^ offering little scope for tuning the spin properties.

In this Article we exploit the chemical control over the coordination sphere available in a family of molecular magnets containing Mn(II) to engineer SECs. Our strategy is to design a molecular geometry, namely [Mn(me_6_tren)*X*]*Y* (where me_6_tren is tris[2-(dimethylamino)ethyl]amine, *X* = Cl, *Y* = ClO_4_ (**1**), *X* = Br, *Y* = PF_6_ (**2**) and *X* = I, *Y* = I (**3**)), which, by virtue of its substantial in-built electric dipole, exhibits a substantial *E*-field-induced deformation that is also coupled to the molecular spin anisotropy. The effect of the electric field in our complexes manifests as a variation of the zero-field splitting (ZFS) parameter *D*, which is the SEC measured in this work. This approach yields molecular SECs comparable with those only observed so far for lanthanide-based molecules with strong SOC.

Our rational design approach allows us to tune the SEC by varying the coordination environment of the spin centre systematically. Wavefunction-based ab initio calculations suggest that the molecular ZFS originates from competing contributions from excited electronic states with distinct symmetries. The large electric dipole along the three-fold symmetry axis allows strong modifications of these contributions by an electric field, leading to substantial SECs. The strongest effect is observed for **3**, where both the electric dipole moment and the *E*-field-induced deformation are the largest.

## Results and discussion

### The Mn(Me_6_tren)*X* compounds

**Fig. 1:**
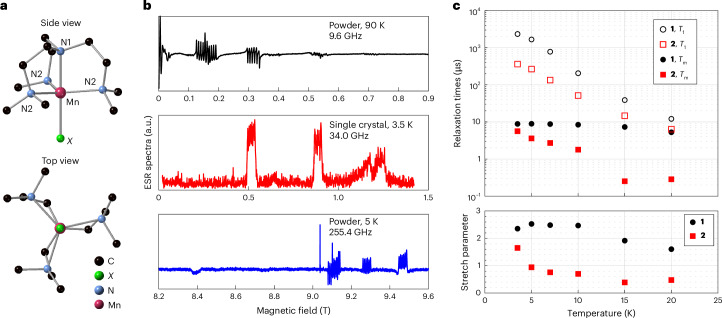
ESR spectra and spin relaxation measurements for 1 and 2. **a**, Ball-and-stick representation of the [Mn(me_6_tren)*X*] molecules. H atoms are omitted for clarity. **b**, Representative low-temperature ESR spectra for **1** recorded with different sample forms at different frequencies. The single-crystal spectrum (middle) was recorded at the Q-band using an echo-detected field sweep (EDFS), whereas the ESR experiments for the powder sample (top and bottom) were conducted using the continuous-wave method. **c**, Low-temperature relaxation times for **1** and **2** molecules measured on the −5/2 ↔ −3/2 and +3/2 ↔ +5/2 transitions, respectively. Upper panel: the spin–lattice relaxation time, *T*_1_, and quantum phase memory time, *T*_m_, for **1** and **2** as a function of temperature. *T*_1_ is described by a single exponential decay over the experimental temperature range. Lower panel: in contrast, *T*_m_ follows a stretched exponential, whose stretch parameter varies with temperature. Source data

The crystallographic structure of Cl derivative **1** is the same as that reported for its Ni(II) and Co(II) counterparts^27–29^. None of the reported Br derivatives with any metal ion crystallize in a trigonal space group. We thus prepared a new compound with Br in the axial position and [PF_6_]^−^ as the counter anion that turned out to crystallize in a trigonal space group. For the I derivative, despite trying several counter anions, they all crystallized in a cubic space group. We therefore prepared the Mn complex based on the reported Zn(II) one, which crystallizes in a cubic space group (Supplementary Tables 1 and 2)^30^. Mn(II) is pentacoordinate, surrounded by one axial (N1) and three equatorial (N2) nitrogen atoms belonging to the tetradentate me_6_tren ligand, and one halogen (*X*). Its coordination sphere has a trigonal bipyramidal (tbp) geometry of *C*_3_ point group symmetry, with the three-fold axis along the N1–Mn–*X* direction (Fig. 1a and Supplementary Fig. 1). The Mn–N1 and Mn–N2 bond lengths and the N1MnN2 angles differ by less than 1% for the three complexes (Supplementary Table 3). The main difference is the Mn–*X* distance: 2.3458(3) Å, 2.5026(18) Å and 2.7133(6) Å, for *X* = Cl, Br and I, respectively. **1** and **2** crystallize in the *R*3*c* and the *R*3*m* trigonal space group, with the *C*_3_ molecular axis along the crystal *c* axis and all the N1–Mn–*X* bonds aligned. **3** crystallizes in the cubic *P*2_1_3 space group with the *C*_3_ molecular axes along the cubic unit cell diagonal. For each compound, we used the corresponding isostructural diamagnetic Zn(II)-containing compounds to provide a diamagnetic host crystal with dilute Mn(II)-complex impurities (Supplementary Sections I and II).

We characterized the magnetic properties using electron spin resonance (ESR) at three frequencies. Representative data for **1** are shown in Fig. 1b (Supplementary Fig. 2 presents more data). The results can be described by an electron spin *S* = 5/2 and a nuclear spin *I* = 5/2 under the Hamiltonian

1 $${\hat{H}}={D{\hat{S}}_{z}^{2}+{\mu }_{\rm{B}}g{{\bf{B}}}_{0}\cdot \hat{{\bf{S}}}+A\hat{{\bf{I}}}\cdot \hat{{\bf{S}}}}$$

where **B**_0_ is the applied magnetic field, *g* and *A* are the isotropic *g*-factor and hyperfine coupling, respectively, and *D* is the axial ZFS parameter. No evidence of a transverse anisotropy was observed for any of the three compounds, consistent with the three-fold rotational symmetry of the molecules. *D* exhibits a systematic trend through the series, with **1** possessing easy-axis-type anisotropy (*D* < 0) and **2** and **3** exhibiting easy-plane-type anisotropy (*D* > 0). By contrast, the hyperfine coupling is almost identical across the family (Table 1).

**Table 1:** Spin Hamiltonian parameters

Molecule	1	2	3
Experiment			
*D* (cm^−1^)	−0.168	+0.188	+0.55
*A* (×10^−3^ cm^−1^)	7.3	7.3	7.1
δ*D*/δ*E* (Hz (V m^−1^)^−1^)	−0.42	−1.20	−1.70
Theory, zero-*E*-field *D*			
*D*_XR_ (cm^−1^)	−0.172	−0.057	+0.098
*D*_DFT_ (cm^−1^)	−0.130	+0.005	+0.174
Theory, δ*D*/δ*E* (Hz (V m^−1^)^−1^)			
(1) electronic effect only	−0.043	−0.112	−0.239
(2) geometry effect only	−0.192	−0.278	−0.498
(3) both effects	−0.234	−0.390	−0.735

The theoretical results for the zero-*E*-field *D* values were calculated using both the X-ray structure (*D*_XR_) and the structure optimized using DFT (*D*_DFT_). δ*D*/δ*E* was calculated using three different cases as described in the main text.

**Fig. 2:**
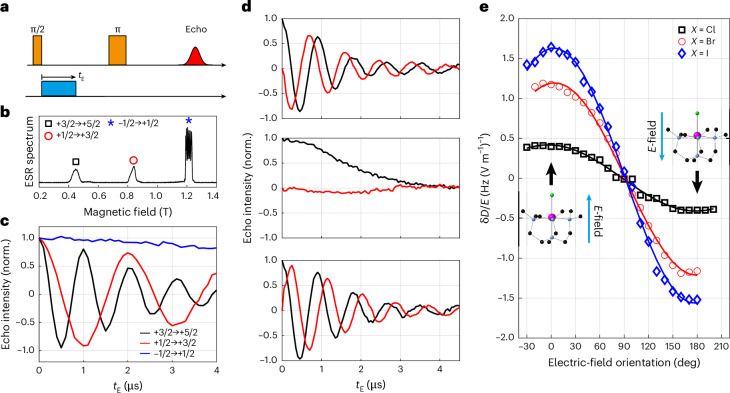
SEC in Mn triangle molecules. **a**, The microwave and *E*-field pulse sequence measuring SEC in single crystals. **b**, The Q-band EDFS spectrum for **2** recorded at 3.5 K. **c**, The in-phase spin echo signals for different *m*_*s*_ transitions as a function of *t*_E_ recorded on **2**. The data were recorded with both *B*_0_ and the pulsed electric field parallel to the Mn–Br bond. **d**, The in-phase (black) and quadrature (red) echo signals for the +3/2 to +5/2 transition in MnBr with the electric field applied parallel (top), perpendicular (middle) and antiparallel (bottom) to the Mn–Br direction. Note that the polarity of the quadrature signal is reversed for the top and bottom data, consistent with a linear SEC. **e**, Orientation dependence of the *E*-field-induced shift in the ZFS parameter *D* (errors are smaller than the symbol sizes). Source data

We measured low-temperature spin relaxation times for **1** and **2** using magnetically diluted single crystals [Mn_0.001_Zn_0.999_(me_6_tren)*X*]*Y* (for **3**, see Supplementary Figs. 3, 4 and 8). The results are shown in Fig. 1 (Supplementary Fig. 4 presents representative relaxation data). The spin lattice relaxation time *T*_1_ for both molecules increases monotonically as the temperature falls, showing no sign of saturation down to our base temperature. At 3.5 K, *T*_1_ for **1** (2.3 ms) is approximately six times that for **2** (0.36 ms). Such a difference in *T*_1_ is probably due to the difference between the Mn–Cl (2.3458(3) Å) and Mn–Br (2.5026(18) Å) bond lengths: the longer Mn–Br distance leads to a weaker bond and lower energy vibrational modes, leading to faster spin–lattice relaxation rates at all temperatures that we studied. This is also consistent with the difference in the ESR spectra for **1** and **2**: although the hyperfine structure of **1** is clearly resolved for all transitions (Supplementary Fig. 2a,e), we could only distinguish it for the *m*_*s*_ = ±1/2 transition in **2** (Fig. 2b and Supplementary Fig. 2b), indicating the presence of a substantial *D* strain.

The phase coherence times (*T*_m_) for **1** and **2** are described by a stretched exponential decay with a temperature-dependent stretch parameter, indicating an interplay between multiple decoherence mechanisms^31^. At 3.5 K, both compounds show similar *T*_m_ values, with their stretched parameters close to 2, suggesting that the decoherence is dominated by the nuclear spin bath surrounding Mn spins. On raising the temperature, the coherence of **2** decreases rapidly, with the stretch parameter dropping below 1 at 5 K. By comparison, both *T*_m_ and the stretch parameter for **1** remain almost temperature-independent up to 10 K (Supplementary Table 4 provides more details).

The average Mn–Mn distance in our 0.1% diluted crystals is less than 8 nm, so electron spin–spin dipolar interactions are of the order of ~1 MHz. The difference in the temperature dependences of *T*_m_ is probably related to the short *T*_1_ associated with **2** (Fig. 1c): *T*_1_ relaxation in **2** leads to magnetic fluctuations in the local environment, inducing contributions to the phase decoherence. Above 10 K, both the *T*_m_ and the stretch parameter for **1** start to decrease rapidly with increasing temperature, suggesting that the nuclear spin bath is no longer the dominant decoherence source.

### SECs

We measured SECs for all three molecules by embedding a square d.c. *E*-field pulse into a Hahn-echo sequence (Fig. 2a and Supplementary Fig. 5). We recorded the spin echo signal as a function of the duration/amplitude of the *E*-field pulse^22,32^ (more details are provided in Supplementary Figs. 6–8). Representative data (recorded on **2**) are shown in Fig. 2. The echo signals for the inter-Kramers transitions exhibit clear oscillations as a function of the duration of the *E*-field pulse (*t*_E_), with the oscillation frequency for the +5/2 ↔ +3/2 signal almost exactly double that for the +3/2 ↔ +1/2 signal. The *E*-field-induced change in the spin transition frequency, δ*f*_E_, is calculated by a fast Fourier transform of the oscillating echo signal. By comparison, the −1/2 ↔ +1/2 transition shows only a weak SEC coupling. Measuring on different hyperfine peaks yields the same weak SEC.

When the magnetic field *B*_0_ is parallel to the magnetic anisotropy axis, the transition within the ±1/2 doublet depends only on *g* and *A*, whereas the inter-Kramers transitions also depend on the ZFS parameter *D*. Hence, the lack of *E*-field dependence for the ±1/2 transition suggests both *A* and *g* show negligible SEC, and the oscillations observed with the inter-Kramers transitions are, therefore, attributable to the *E*-field modulation of the anisotropy parameter *D*. This is further supported by the fact that the ZFS for the +5/2 ↔ +3/2 transition, 4*D*, is exactly twice that for the +3/2 ↔ +1/2 transition, 2*D*. Accordingly, an *E*-field-induced modification in *D*, δ*D*, lead to oscillation in the +5/2 → +3/2 echo at frequency 4δ*D*, double that for the +3/2 → +1/2 echo, 2δ*D*.

Both **1** and **2** crystallize in a polar space group with all molecules co-aligned. Consequently, all molecules should exhibit the same linear response upon application of an external *E*-field, allowing us to measure both the amplitude and sign of δ*f*_E_. (The sign of *δf*_E_ is inaccessible for random orientated samples, such as frozen solutions^32^ or single crystals with inversion-related molecules^22^.) The in-phase and quadrature parts of the echo signal should follow cos(2πδ*f*_E_*t*_E_) and sin (2πδ*f*_E_*t*_E_), respectively, where the sign of δ*f*_E_ is determined by the polarity of the quadrature component, as illustrated in Fig. 2d (more details are provided in Supplementary Figs. 6–8). When the orientation of the electric field is rotated from the Mn–Br (top) to the Br–Mn (bottom) direction, the quadrature part of the signal is inverted, whereas the in-phase part remains virtually identical, as expected for a linear SEC.

The full orientation dependence of the SECs is mapped by rotating the *E*-field against the crystals. We present the *E*-field-induced changes in *D* for all three molecules for direct comparison (Fig. 2e). For all molecules the maximum SECs occur with the applied electric field parallel or antiparallel to the Mn–*X* bond, with a near-complete extinction of the effect for the perpendicular orientation. This highlights the importance of the molecular electric dipole: an *E*-field is coupled to the molecular spin via its electric dipole. Hence, even though the triangular plane perpendicular to the Mn–*X* bond also does not possess an inversion symmetry, allowing first-order SEC^6,33,34^ by symmetry, an *E*-field applied in this plane cannot couple to the spin efficiently due to the lack of an electric dipole in this orientation.

The observed *E*-field-induced frequency shifts (~4.8 Hz (V m^−1^)^−1^ for **2**) are substantial, especially considering that Mn(II) ions are typically associated with a weak spin–orbit interaction due to their half-filled 3*d*^5^ outer shell. The coupling to the spin spectrum (δ*f*_E_/*E*) is much stronger than that for molecular magnets containing Mn(II) (~0.68 Hz (V m^−1^)^−1^)^24^ and comparable with the shift for a lanthanide-based molecule (~11 Hz (V m^−1^)^−1^) with giant SOC^22^. The *E*-field effect on the ZFS parameter, δ*D*/*E* = 1.7 Hz (V m^−1^)^−1^ for **3**, is also comparable to those found for Mn^2+^ spins doped in inorganic crystals: 1.26, 2.25, 1.33 and 3.3 Hz (V m^−1^)^−1^ for CaWO_4_ (ref. ^35^), SrWO_4_ (ref. ^36^), PbMoO_4_ (ref. ^37^) and ZnO (ref. ^26^), respectively. For the scheelite lattices^35–37^, the SEC is largely attributed to the displacement of the spin-carrying ion in the applied electric field. In ZnO (ref. ^26^), the SEC is associated with the piezoelectric nature of the host lattice, allowing a substantial *E*-field-induced distortion of the lattice geometry. These previous works are consistent with our findings: a strong SEC is probably due to the substantial molecular electric dipole and the fact that it is directly correlated to the molecular magnetic anisotropy. An *E*-field distorts the geometry of the molecule, modulating the ZFS of the Mn^2+^ spins.

The SECs for the complexes could be adequate for practical spin control with an *E*-field generated by sufficiently localized electrodes. For example, with a d.c. *E*-field of 10^7^ V m^−1^, that is, 10 mV nm^−1^, the resonance frequency of **3** can be shifted by ~68 MHz, corresponding to the excitation bandwidth of a 15-ns microwave pulse. A high-frequency resonance a.c. *E*-field of the same amplitude can drive coherent spin transitions with the Rabi rate of ~15 MHz and ~1.7 MHz for standard δ*m*_s_ = 1 and double δ*m*_s_ = 2 transitions, respectively (applying *B*_0_ perpendicular to the anisotropy axis), potentially allowing hundreds of coherent *E*-field-driven spin operations within *T*_m_. *E*-fields on this scale are routinely accessible in reported molecular break-junction devices^7,38^ and scanning tunnelling microscopy experiments^11^.

Despite the fact that *D* < 0 for *X* = Cl and *D* > 0 for *X* = Br and I, we note that δ*D* < 0 for all three compounds when an electric field is applied pointing from the *X* halogen ion towards Mn^2+^ (Supplementary Table 5). Such behaviour showcases the possibility of controlling magnetic anisotropy and SEC independently, allowing the design of molecular magnets with strong SEC while maintaining operability within the microwave frequency range convenient for (quantum) information technologies. This can be understood qualitatively by considering the origin of the magnetic anisotropy and the symmetry of their electronic states (see next section).

### Electronic structure calculations

We performed wavefunction-based ab initio calculations to understand the origin of *D* and its interaction with an external *E*-field^10,39–41^. The ZFS parameters for all molecules (without external *E*-field) were calculated using two geometries: the X-ray structures and the molecular geometries optimized in density functional theory (DFT) while preserving *C*_3_ symmetry. Both calculations reproduce the trend of *D* observed in ESR measurements; that is, the ZFS shifts from easy-axis type (*D* < 0) to easy-plane type (*D* > 0) as the halogen atom varies from Cl to I. Here we focus on results obtained using the DFT-optimized geometry, as this allows us to investigate the *E*-field-induced distortions to the geometry of the molecules.

Detailed analysis was performed with **1** and **3** to rationalize the origin of the ZFS. For a high-spin Mn(II) (*S* = 5/2) ground state, all five *d* orbitals are singly occupied, leading to a sextuplet ground state ^6^*A*. Therefore, the ZFS can only emerge due to interactions between the electronic ground state and the excited quadruplet states, ^4^*Y*^*i*^, via SOCs. It is worth noting that the spin–spin contribution to *D* (ref. ^42^), which is considered in the calculations, is very small.

For analysis purposes, we can consider the second-order perturbation expression of the SOC contributions. The SOC interaction between the ground electronic state ${{| }^{6}{A}_{{m}_{\rm{s}}}\left.\right\rangle}$ with the spin projection *m*_s_ and the *m*_sl_ component in an excited electronic state ^4^*Y*^*i*^ leads to a contribution to *D* of the ground state, ${c(D)[{4\atop}{Y}_{{m}_{\rm{sl}}}^{\,i}]}$:

2 $${c(D)\left[{4\atop}{Y}_{{m}_{\rm{sl}}}^{i}\right]}={\sum _{k}\frac{\left| \left\langle {6\atop}{A}_{{m}_{\rm{s}}}| {\zeta }_{k}\left[\left({\hat{L}}_{k}^{+}{\hat{S}}_{k}^{-}+{\hat{L}}_{k}^{-}{\hat{S}}_{k}^{+}\right)/2+{\hat{L}}_{k}^{z}{\hat{S}}_{k}^{z}\right]| {4\atop}{Y}_{{m}_{\rm{sl}}}^{\,i}\right\rangle \right| ^{2}}{{\mathcal{E}}\left({4\atop}{Y}^{i}\right)}}$$

where the sum runs over all electrons *k* of the *d* shell. ${\mathcal E} ({4\atop}{Y}^{\,i})$ is the energy of the ^4^*Y*^*i*^ excited state with respect to the ground state and *ζ*_*k*_ is the SOC constant, which depends on the two orbitals involved in the excitation. By summing over the *m*_s_ and *m*_sl_ components of both the ground and excited states, one obtains the full contribution *C*(*D*) of each excited state. The sum of the contributions of all ^4^*Y*^*i*^ excited states, ∑*C*(*D*), leads to the ZFS. Ab initio calculations show that the main contributions to *D* arise from the ten excited quadruplet states. Among them, four doubly degenerate states *E*^*i*^ (*i* = 1 to 4) that couple to the ground state through the $({\hat{L}}_{k}^{+}{\hat{S}}_{k}^{-}+{\hat{L}}_{k}^{-}{\hat{S}}_{k}^{+})/{2}$ term lead to negative contributions to *D*, and the two non-degenerate states *A*^*i*^ (*i* = 1 or 2) that couple to the ground state through ${\hat{L}}_{k}^{\,z}{\hat{S}}_{k}^{\,z}$ lead to positive contributions to *D* (ref. ^43^).

The excitation energies are driven by the ligand field and follow the halogen spectrochemical series (Supplementary Fig. 9 and Supplementary Section V.A). However, although for many series of complexes the excitation energies govern the magnitude and nature of *D*, here the variation of the SOCs plays the most important role. Indeed, one may notice that the increase or decrease in the contributions to *D* of an excited state is directly correlated with the decrease or increase of the SOCs (Supplementary Table 6). The variation in SOCs can have two origins: either the coefficient on the ^4^*Y* state determinants involved in the coupling varies between **1** and **3**, or the spin–orbit constants *ζ*_*k*_ vary. In the present case, both variations need to be considered. However, the dominant effect concerns the spin–orbit constants. Indeed, for an excitation involving an orbital with a *z* component (that is pointing towards the halogen), the constant *ζ*_*k*_ is weaker for the iodine-containing complex than for the chlorine-containing one due to the relativistic nephelauxetic effect, inducing weaker couplings and therefore lower negative contributions. Concerning the *A*^2^ state, it is essentially carried by the two excitations from *d*_*xy*_ to ${d}_{({x}^{2}-{y}^{2})}$ (Supplementary Table 7) and vice versa, and the weight on these two configurations (Supplementary Table 8) is larger in **3** than in **1**, inducing a stronger coupling and therefore a larger positive contribution. To summarize, the negative contributions to *D* due to the quadruplet *E*^*i*^ states decrease from **1** to **3**, whereas the positive contributions brought by the *A*^*i*^ states increase, resulting in an overall ZFS shifting from easy-axis to easy-plane type, as experimentally observed.

**Fig. 3:**
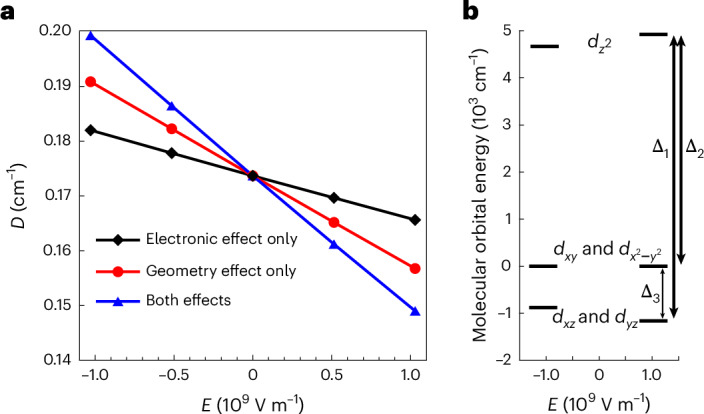
Theoretical calculations for 3. **a**, Theoretical calculation for **3** showing a linear SEC. A positive *E* corresponds to an *E*-field applied from I^−^ to Mn^2+^. The calculations were performed with three cases as described in the main text. **b**, Molecular orbital energy diagram for **3** with the application of an *E*-field. Source data

The application of an *E*-field modifies both the electronic structure and the geometry of the molecules, thus changing *D*. To appreciate the spin–electric effect due to each contribution individually, we calculated *D* using the following three cases: (1) an *E*-field only modifies the electronic structure, with the molecular geometry unperturbed; (2) an *E*-field distorts the geometry of the molecule, leading to a new structure (optimized using DFT in the presence of the *E*-field) with which *D* is calculated; (3) *D* is calculated using the new geometry in the presence of an *E*-field affecting the electronic structure. To reduce relative digital errors in the ab initio calculation, a strong electric field is used (~10^9^ V m^−1^), substantially larger than those applied in experiments (~10^5^ V m^−1^). Nevertheless, the calculations produce a linear *E*-field dependence of *D* (Fig. 3a), allowing us to draw a direct comparison between calculations and experiments.

Representative results for **3** are shown in Fig. 3 (Supplementary Fig. 10 presents results for **1** and **2**). When an *E*-field is applied pointing from the halogen ion towards the Mn(II) ion, it distorts the molecular geometry such that the Mn–*X* distance increases (Supplementary Table 9) while the Mn–N bond length decreases. This changes the electronic structure of the molecule such that all energy differences between the orbitals increase, as shown in Fig. 3b. Note that this increase is larger in the iodine-containing complex than in the chlorine one due to the larger polarizable character of iodine; that is, a stronger deformation of the electronic cloud induces a larger geometric distortion of the molecule. More importantly and as explained in the Supplementary Information (Supplementary Tables 10–13), the application of an *E*-field varies the SOCs, leading to a weaker *D* > 0 contribution by the *A*_*i*_ states and a stronger *D* < 0 contribution by the *E*_*i*_ states. These two modulations combine constructively and give rise to the overall *E*-field-induced modulation of *D*.

The results are summarized in Table 1. The calculations successfully reproduce the trend observed in experiments, with increasing effects when the halogen is changed from Cl to I. The variations in dipole moment also follow the expected trends (Δ*μ*_e_ = 2.063 D, 2.194 D and 2.465 D for **1**, **2** and **3**, respectively; Supplementary Table 14); that is, a positive (negative) *E*-field increases (decreases) the dipole moment. As the geometric distortions follow the displacements of the electron cloud, the two effects in cases (1) and (2) add up almost perfectly (Supplementary Table 14). The comparison of the dipole moments in the spectrochemical series also shows that the *E*-field-induced effect is stronger for **3** than that for **1**, as I^−^ is more polarizable than Cl^−^, so applying an *E*-field leads to a larger distortion in **3** and a stronger modulation of *D*, despite the SOC constant being stronger for **1**.

To check that the effect of the electric field on the crystallographic structures follows the same trend as on the optimized ones, we performed the same analysis (case (1)) on the experimental structures of **1** and **3** (Supplementary Tables 15 and 16) and found the same results: the variation of the contributions to *D* follows the variations of the SOCs and can therefore be attributed to changes in the ground- and excited-state wavefunctions. Overall, we can conclude that the structural distortions follow the field-induced deformations of the electron cloud and are therefore stronger for **3** than for **1**, as I^−^ is more polarizable than Cl^−^. Crucially, our analysis reveals that the distortions to the molecular geometry play the major role for all three molecules^22^.

Finally, we note that, compared to the optimized structures used in the calculation, which are obtained considering single molecules in vacuum, the actual crystal structure contains counterions that can lead to larger distortions. Therefore, it is conceivable that the calculations underestimate the electric-field effect. Nevertheless, the theoretical results are in reasonable agreement with the experimental data.

## Conclusions

Our main findings are that it is possible to control the ZFS with an electric field through a spin–electric effect by prudent design of molecular complexes, and that we can generate a substantial spin–electric effect without involving a strong SOC. The theoretical analysis showcases the importance of geometry distortions in driving the spin–electric effect, and the possibility of harnessing the competing interactions to tune the ZFS and spin–electric effect independently.

Based on these findings, we propose that a large SEC would be obtained either by (1) increasing the response to the electric field, that is, by choosing highly polarizable ligands likely to induce a large dipole moment, or (2) increasing SOCs, that is, by involving transition-metal ions possessing an odd number of electrons in degenerate orbitals (either the orbitals ${d}_{{x}^{2}-{y}^{2}}$ and *d*_*xy*_ or *d*_*xz*_ and *d*_*yz*_), which generates a sizable unquenched orbital momentum. This is achievable by choosing ligands that impose axial symmetry (*C*_*∞*_, *C*_3_ or *C*_5_ for instance)^27,44–46^. To be used as electrically addressable quantum bits, an ESR transition in the microwave energy domain sensitive to the electric field is needed. Our work suggests routes to QIP technologies based on molecular design principles, yielding electrically addressable molecular qubits compatible with current microwave techniques.

## Methods

### Continuous ESR studies

Continuous-wave (c.w.) X-band ESR spectra were recorded on a Bruker ELEXSYS 500 spectrometer equipped with a Bruker ER 4116DM X-band resonator and an Oxford Instruments continuous-flow ESR 900 cryostat and ITC 503 temperature-control system. The experimental conditions were as follows: microwave frequency = 9.636 GHz, microwave power = 4.0 mW, modulation amplitude = 8 G, modulation frequency = 100 kHz, gain = 34 dB, temperature = 90 K, number of scans = 2.

High-frequency ESR (HF-ESR) spectra were recorded on a multifrequency spectrometer operating in a double-pass configuration^47^. Frequencies were obtained with a 128 GHz source (Virginia Diodes) associated to a doubler or with a 110 GHz source (Virginia Diodes) associated to a tripler. The exciting light was propagated with a Quasi-Optical set-up (Thomas Keating) outside the cryostat and with the help of a corrugated waveguide inside it. Detection was performed with a hot electron InSb bolometer (QMC Instruments). The main magnetic field was supplied by a 16 T superconducting magnet associated to a VTI (Cryogenic). Derivative spectra were obtained with a field modulation amplitude of 1.7 G. The measurements were performed on powdered samples pressed into pellets to limit torqueing effects. The simulated spectra were obtained with the SIM program^48^ from H. Weihe (Univ. Copenhagen), through an ‘error and trial’ process without fitting. A simple Lorentzian line shape was used in the simulation with a fixed intrinsic linewidth of 20 G for the ESR transitions in all three complexes.

### Spin coherence and SEC studies

Pulsed ESR measurements were performed to investigate the spin coherence and SEC in the compounds. The experiments were conducted on a Bruker E580 c.w./pulsed spectrometer. The Q-band (~34 GHz) experiments were performed using a Bruker ER 5106 QT-W probe with a maximum sample access of 3 mm. This Q-band probe, though nominally designed for c.w. measurements, can be used for pulsed ESR experiments^49^. We chose to conduct Q-band pulsed ESR measurements using this probe, instead of dedicated pulsed probes such as the Bruker EN5107D2, to provide sufficient access space for the SEC apparatus. The X-band (~9.7 GHz) experiments were performed using a Bruker ER 4118X-MD5-w1 c.w./pulsed probe with a maximum sample access of 5 mm. High-power microwave pulses were obtained using travelling-wave tube (TWT) amplifiers. The peak power of the Q-band and the X-band TWT amplifiers were ~140 W and ~1 kW, respectively. An Oxford Instruments continuous-flow ESR CF935 cryostat and ITC 503 temperature-control system were used for temperature control. For all pulsed experiments presented in this work, we used 16-ns and 32-ns durations for π/2 and π pulses, respectively, to keep the excitation bandwidth consistent throughout the study, and adjusted only the amplitude of the pulses. This corresponds to a microwave *B*_1_ field of ~5 G.

The SECs for all three molecules (**1**, **2** and **3**) were measured by embedding a square d.c. *E*-field pulse into a Hahn-echo sequence (Supplementary Fig. 6d). The microwave pulse sequence parameters (microwave pulse amplitude, duration and separation *τ*) remained unchanged in the experiments, and the spin echo signal was recorded as a function of the duration/amplitude of the *E*-field pulse^22,32^. The experimental apparatus is similar to the one reported in ref. ^22^ with minor modifications to accommodate for the smaller sample space of the Q-band probe. A pair of conductive plates (2 × 50 mm^2^), separated by 2 mm, were inserted into the microwave resonator for application of the electric field. The conductive plates were prepared by electron-beam evaporation of 10 nm of titanium and 100 nm of gold onto 200-μm-thick bare printed circuit boards made of FR4. The thickness of the conductive layer was chosen to ensure good electric conductivity while minimizing the perturbation to the microwave resonator mode. Both conductive plates were glued to a 2-mm-thick spacer (made of FR4; Supplementary Fig. 5a). The spacer separated the two conductive plates as well as providing mechanical support. The conductive plates were connected to a rigid coaxial cable. One plate was connected to the metal shield of the coaxial cable, which was the electrical ground. This connection also provided mechanical support to the plates. The second plate was electrically connected to the centre core of the cable, allowing application of voltage (*E*-field) pulses. The *E*-field pulse was generated by applying voltage pulses using an AVTECH AVR-4-B pulse generator with a typical rising/falling edge of 15 ns and maximum output of 400 V. A 50-Ω matching resistance was also connected to the electric circuit to mitigate *E*-field ringing at the sample. More details of the spin coherence and SEC studies are included in Supplementary Section IV.

### Computational studies

The geometries of the *m*_s_ = 5/2 solution of the complexes were optimized by imposing the *C*_3_ axis both with and without the application of an electric field using DFT (with the wB97X-D3 function)^50^ to assess the impact of the applied field on the structures. Complete active space self-consistent field (CASSF) calculations, followed by *n*-electron valence-state perturbation theory (NEVPT2)^51–53^ treatment of dynamical correlations, were used to describe spin–orbit free states. The active space contained all *d* electrons in all *d* orbitals, that is, CAS(5,5), and the orbitals were averaged for the sextuplet ground state and the 24 excited quadruplet states of the configuration with an equal weight on the *S* = 5/2 state (50%) and on the *S* = 3/2 all together (50%). Finally, the spin–orbit state interaction method^54^ was used to treat relativistic effects with the ZORA Hamiltonian^55^. ZFS parameters were extracted using effective Hamiltonian theory^56,57^, according to a method that has been successfully applied to many transition-metal complexes^39^, including rationalization of the impact of an electric field on anisotropic spin Hamiltonian parameters^10,40,41^. The same atomic basis sets were used for DFT and CASSCF+NEVPT2 calculations: ZORA-def2-TZVPP for Mn, N, Cl, Br atoms^58^, SARC-ZORA-TZVPP for the I atom^59^, ZORA-def2-TZVP for C atoms^58^, and ZORA-def2-SVP for H atoms^58^. Autoaux auxiliary basis sets were also used for efficiency reasons^60^. All results were obtained by mean of the standard code ORCA5.0^61^.

## Online content

Any methods, additional references, Nature Portfolio reporting summaries, source data, extended data, supplementary information, acknowledgements, peer review information; details of author contributions and competing interests; and statements of data and code availability are available at 10.1038/s41557-025-01926-5.

## Supplementary information

Supplementary Information Supplementary Figs. 1–10, Discussion and Tables 1–16.

Supplementary Table The atomic coordinates of all the optimized models used in this work.

## Source data

Source Data Fig. 1 ESR spectra and spin relaxation measurements.

Source Data Fig. 2 ESR spectrum for compound 2 and spin–electric coupling measurements.

Source Data Fig. 3 Theory for spin–electric coupling for compound 3 and E-field dependence of orbital energy.

## Data Availability

All data supporting the findings of this study are available within this Article and its Supplementary Information. The ESR data, the SEC data and the coordinates of the calculated structures have been deposited to figshare with the dataset identifier 10.6084/m9.figshare.28541423 ref. ^62^. Data are also available from the corresponding authors upon reasonable request. Crystallographic data for structures reported in this Article have been deposited at the Cambridge Crystallographic Data Center (CCDC) under deposition nos. CCDC 2152258 (**1**), 2152257 (**2**), 2152259 (**3**), 2270433 (**4**) and 2270432 (**5**). Copies of the data can be obtained free of charge via https://www.ccdc.cam.ac.uk/structures/. Source data are provided with this paper.
