# Supplementary material for: Chemical tuning of quantum spin–electric coupling in molecular magnets
Source: Nat Chem. 2025 Aug 27;17(12):1903–9. doi: 10.1038/s41557-025-01926-5 (PMC12669029; doi:10.1038/s41557-025-01926-5)
Supplement: Supplementary file 1 — Supplementary Figs. 1–10, Discussion and Tables 1–16. [file 41557_2025_1926_MOESM1_ESM.pdf]

nature chemistry

Supplementary information

https://doi.org/10.1038/s41557-025-01926-5

Chemical tuning of quantum spin–electric coupling in molecular magnets

In the format provided by the authors and unedited

Contents

I. Synthesis 2
A. Synthesis of [Mn(Me_6tren)Cl](ClO_4) (**1**) 2
B. Synthesis of [Mn(Me_6tren)Br](PF_6) (**2**) 2
C. Synthesis of [Mn(Me_6tren)I]I (**3**) 2
D. Synthesis of Zinc(II) derivatives 2

II. Crystallography data for the molecules 3

III. Continuous-wave electron spin resonance measurements 5

IV. Pulsed electron spin resonance and measurements for spin-electric couplings 7
A. Spin coherence measurements 7
B. Spin-electric coupling measurements 9

V. Computational details 13
A. The ZFS parameter for the [Mn(me_6tren)X]Y_2 molecules 13
B. Spin-electric coupling for the [Mn(me_6tren)X]Y_2 molecules 15

References 19

I. Synthesis

Chemicals were purchased from Aldrich and used without further purification. Me6tren and was synthesized according to literature procedure. All solvents were used as received. All manipulations were conducted under standard benchtop conditions. Hazardous: perchlorate salts are prone to explode, they must be manipulated with care and always using small quantities. The solid compound must not be heated or put under sudden pressure.

A. Synthesis of [Mn(Me$_6$tren)Cl](ClO$_4$) (1)

$MnCl_2 \cdot 4H_2O$ (0.197 g, 1mmol) was dissolved in degassed EtOH (100 ml) at 55 °C. Me6tren (0.230 g, 1 mmol) dissolved in degassed EtOH (10 ml) was added dropwise with stirring for few minutes. $NaClO_4 \cdot H_2O$ (0.280 g, 2 mmol) dissolved in degassed EtOH (20 ml) was added with stirring inducing the precipitation of a white powder: [Mn(Me$_6$tren)Cl](ClO$_4$) (**1**) (0.39 g, yield 93%), which was isolated by filtration and dried in air. Crystals suitable for X-ray analysis were formed upon diffusion of diethylether in acetonitrile solution of **1**. Crystals were slightly sensitive to oxidation if left a long time in aerobic atmosphere, they were stored under Argon.

- Elemental analysis (%), observed (calculated for $MnC_{12}H_{30}N_4Cl_2O_4$) C, 34.47 (34.00) H, 7.03 (7.20) N, 13.28 (13.30).
- MS ESI$^+$ (MeOH): m/z 320.16, 321.15, 322.15, 323.15 [Mn(Me$_6$tren)Cl]$^+$. Calculated for (C12H30N4MnCl) 320.15, 321.15, 322.15, 323.15
- IR (KBr) v/cm^{-1}: 3400 (large w), 2976 (m), 2885 (m), 1485 (s), 1360 (w), 1304 (s), 1246 (w), 1173 (m), 1090 (vs), 1026 (s), 1023 (m), 1006 (m), 934 s), 905 (m), 800 (m), 773 (s), 622 (s), 473 (w).

B. Synthesis of [Mn(Me$_6$tren)Br](PF$_6$) (2)

The same procedure as for **1** was used to prepare **2** starting from $MnBr_2 \cdot 4H_2O$ and using NH_4PF_6 instead of $NaClO_4 \cdot H_2O$. The crystals were slightly sensitive to oxidation if left a long time in aerobic atmosphere, they were stored under Argon.

- Elemental analysis (%), observed (calculated for $MnC_{12}H_{30}N_4BrPF_6$) C, 28.31 (28.25) H, 5.78 (5.93) N, 10.92 (10.98).
- MS ESI$^+$ (MeOH): m/z 364.10 [Mn(Me$_6$tren)Br]$^+$.
- IR (KBr) v/cm^{-1}: 3011 (w), 2980 (m), 1484 (s), 1473 (s), 1439 (m), 1414 (w), 1359 (w), 1303 (s), 1245 (w), 1174 (m), 1105 (s), 1055 (m), 1045 (m), 1027 (m), 935 (m), 905 (m), 838 (vs), 799 (s), 771 (s), 595(w), 557 (s), 471 (w).

C. Synthesis of [Mn(Me$_6$tren)I]I (3)

3 was prepared by a adapting the procedure reported on the Zn(II) derivative [1]. MnI_2 was prepared from Mn powder and I_2 in THF as follows: 0.12 g (2.2 mmol) of Mn powder was added to 40ml of THF in a Schlenk flask under argon atmosphere. Then 0.51 g (2 mmol) of I2 dissolved in 40 ml of THF was added to the previously prepared suspension that was stirred at RT for 24 h. After filtration under argon the clear orange THF solution of MnI_2 was used directly for the for the preparation of **3** by adding a degassed THF solution of Me6tren (0.214 g, 0.92 mmol, 50 ml). After 10 min of stirring, the precipitate was filtered under argon: (0.26 g, yield 75%), The microcrystalline powder was dissolved with a minimum of acetonitrile. By diffusion of diethyl ether in to the acetonitrile solution, well-shaped crystals were obtained. They were stored under argon to prevent possible oxidation.

- IR (KBr) v/cm-1: 3400 (large, w), 3008 (m), 2979 (m), 2966 (m), 2869 (m), 2802 (w), 1471 (s), 1454 (s), 1354 (m), 1294 (w), 1274 (m), 1170 (w), 1101 (s), 1017 (m), 1033 (m), 988 (s), 932 (s), 907 (s), 797 (s), 769 (s), 596 (w), 476 (m).

D. Synthesis of Zinc(II) derivatives

The Zn(II) derivatives were prepared by the same procedure as for the Mn(II) complexes. Elemental analysis (%) for the three compounds is given below:

- [Zn(Me_6tren)Cl](ClO_4) (**4**): observed (calculated for $ZnC_{12}H_{30}N_4Cl_2O_4$) C, 33.73 (33.47) H, 6.86 (7.20) N, 12.9 (13.01)
- [Zn(Me_6tren)Br](PF_6) (**5**): observed (calculated for $ZnC_{12}H_{30}N_4BrPF_6$) C, 28.73 (27.68) H, 5.91 (5.81), N 10.73 (10.76)
- [Zn(Me_6tren)I]I (**6**): observed (calculated for $ZnC_{12}H_{30}N_4I_2$) C, 26.43 (26.23) H, 5.29 (5.50), N 10.17 (10.19)

The diluted complexes 1% and 0.1% of Mn in the diamagnetic Zn matrix were obtained by performing the same synthetic procedure for each compounds introducing 1% and 0.1% of the Mn starting materials. The only test that attests that dilution was effective is the cw X-band ESR spectra, where we could see that the 0.1% complexes had their intensities that were reduced by roughly one order of magnitude in comparison to the 1% concentrated samples. The cw X-band spectra of the pure compounds show only large bands without any hyperfine structure. Due to the lack of sufficiently sensitive techniques, no further analysis was conducted to accurately determine the material concentrations.

II. Crystallography data for the molecules

Supplementary Table 1. **Crystallographic data and structure refinement details for compounds 1, 2 and 3.**

Compound	[Mn(Me_6trenCl)]ClO_4 (**1**)	[Mn(Me_6trenBr)]PF_6 (**2**)	[Mn(Me_6trenI)]I (**3**)
CCDC	2152258	2152257	2152259
Empirical formula	C_{12} H_{30} Cl Mn N_4, Cl O_4	C_{12} H_{30} Br Mn N_4, P F_6	C_{12} H_{30} I Mn N_4, I
M_r	420.24	510.22	539.14
Crystal size, mm^3	0.38 × 0.28 × 0.21	0.13 × 0.09 × 0.03	0.19 × 0.17 × 0.11
Crystal system	trigonal	trigonal	cubic
Space group	$R3c$	$R3m$	$P2_13$
a (Å)	10.0600(3)	10.4743(4)	12.6201(7)
b (Å)	10.0600(3)	10.4743(4)	12.6201(7)
c (Å)	32.5354(10)	16.0523(6)	12.6201(7)
$\alpha(°)$	90	90	90
$\beta(°)$	90	90	90
$\gamma(°)$	120	120	90
Cell volume ($Å^3$)	2851.56(19)	1525.17(13)	2010.0(3)
Z; Z'	6; 1/3	3; 1/6	4; 1/3
T (K)	100(1)	200 (1)	200(1)
Radiation type; wavelength (Å)	Mokα; 0.71073	Mokα; 0.71073	Mokα; 0.71073
F_{000}	1326	777	1044
μ, mm^{-1}	0.998	2.749	3.724
range (°)	2.652 - 45.391	2.579 - 30.560	2.282 - 32.546
Reflection collected	43 883	9 341	29 158
Reflections unique	5 134	959	2 045
R_{int}	0.0238	0.0395	0.0327
GOF	1.076	1.163	1.107
Refl. obs. ($I > 2(I)$)	4 473	946	1 898
Parametres	105	70	61
wR_2 (all data)	0.0460	0.0873	0.0264
R value ($I > 2(I)$)	0.0178	0.0320	0.0152
Largest diff. peak and hole (e.$Å^{-3}$)	0.369 ; -0.366	0.637 ; -0.580	0.313 ; -0.332

Supplementary Table 2. **Crystallographic data and structure refinement details for the diamagnetic Zn molecules.**

Compound	[Zn(Me$_6$trenBr)]PF$_6$ (**5**)	[Zn(Me$_6$trenCl)]ClO$_4$ (**4**)
CCDC	2270432	2270433
Empirical formula	C_{12} H_{30} Br N_4 Zn, P F_6	C_{12} H_{30} Cl N_4 Zn, Cl O_4
M_r	520.65	430.67
Crystal size, mm^3	0.11 × 0.07 × 0.04	0.18 × 0.17 × 0.08
Crystal system	trigonal	trigonal
Space group	$R3m$	$R3c$
a (Å)	10.4051(8)	9.9059(5)
b (Å)	10.4051(8)	9.9059(5)
c (Å)	15.9295(12)	33.1244(19)
$\alpha(^\circ)$	90	90
$\beta(^\circ)$	90	90
$\gamma(^\circ)$	120	120
Cell volume (Å^3)	1493.6(3)	2814.9(3)
Z; Z'	3; 1/3	6; 1/3
T (K)	100(1)	100 (1)
Radiation type; wavelength (Å)	Mokα; 0.71073	Mokα; 0.71073
F_{000}	792	1356
μ, mm^{-1}	3.377	1.616
range (°)	2.597 - 30.546	2.674 - 38.722
Reflection collected	15 941	54 387
Reflections unique	1 168	3 548
R_{int}	0.0565	0.0564
GOF	1.089	1.061
Refl. obs. ($I > 2(I)$)	1 159	3 046
Parametres	70	88
wR_2 (all data)	0.0522	0.0474
R value ($I > 2(I)$)	0.0213	0.0200
Largest diff. peak; hole (e.Å^{-3})	0.315 ; -0.605	0.275 ; -0.517

X-ray diffraction data for compounds [Mn(Me$_6$trenCl)]ClO$_4$ (**1**), [Mn(Me$_6$trenBr)]PF$_6$ (**2**) and [Mn(Me$_6$trenI)]I (**3**) were collected by using a Kappa X8 APPEX II Bruker diffractometer with graphite-monochromated MoKα radiation (λ = 0.71073 Å). X-ray diffraction data for the diamagnetic version of the compounds, [Zn(Me$_6$trenCl)]ClO$_4$ and [Zn(Me$_6$trenBr)]PF$_6$, were collected by using a VENTURE PHOTON 100 CMOS Bruker diffractometer with Micro-focus IuS source MoKα radiation. Crystals were mounted on a CryoLoop (Hampton Research) with Paratone-N (Hampton Research) as cryoprotectant and then flashfrozen in a nitrogen-gas stream at 100K or 200K. For compounds, the temperature of the crystal was maintained at the selected value by means of a 700 series Cryostream cooling device with an accuracy of ±1 K. The data were corrected for Lorentz polarization, and absorption effects. The structures were solved by direct methods using SHELXS-97 [2] and refined against F^2 by full-matrix least-squares techniques using SHELXL-2018 [3] with anisotropic displacement parameters for all non-hydrogen atoms. All calculations were performed by using the Crystal Structure crystallographic software package WINGX [4]. The crystal data collection and refinement parameters are given in Table 1 and 2.

CCDC 2152257-2152259 and 2270432-2270433 contains the supplementary crystallographic data for this paper. These data can be obtained free of charge from the Cambridge Crystallographic Data Centre: http://www.ccdc.cam.ac.uk/Community/.

The crystals' faces used for the single crystal EPR experiments under the effect of the electric field were indexed prior to the experiments in order to determine the orientation of the Mn-X direction with respect to the crystals c axis that coincides with this direction for the Cl and Br derivatives. For the I derivative, the crystal was also oriented

and the electric field was applied as shown in Fig. 8 (see below).

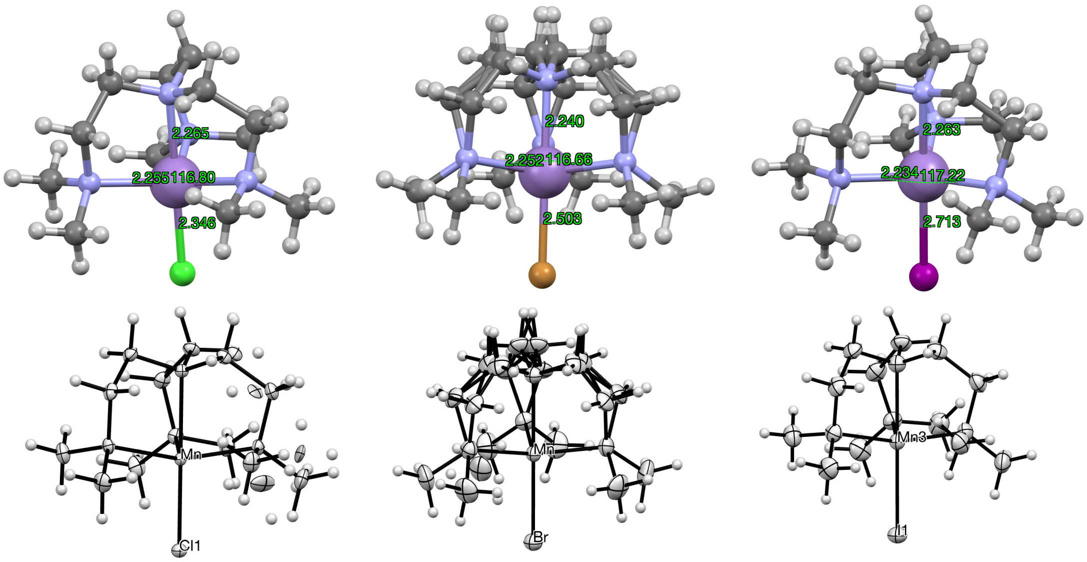

Supplementary Fig. 1. **Molecular structures of compounds 1, 2 and 3.** top: View of the molecular structures of $[\mathrm{Mn(me_6tren)}X]^+$ with X = Cl (left), Br (middle) and I (right). bottom: View of the molecular structures with ORTEP representation with X = Cl (left), Br (middle) and I (right).

Supplementary Table 3. **Bond distance and angles for the Mn coordination sphere.** All esds are estimated using the full covariance matrix.

	Mn-N_{ax} (Å)	Mn-N_{eq} (Å)	Mn-X (Å)	N_{ax}-Mn-N_{eq} (°)
**1**	2.2648(12)	2.2552(6)	2.3458(3)	79.573(18)
**2**	2.240(11)	2.255(5)	2.5026(18)	79.37(15)
**3**	2.263(3)	2.2344(18)	2.7133(6)	80.30(5)

III. Continuous-wave electron spin resonance measurements

High-frequency ESR (HF-ESR) measurements were performed to determine the magnetic anisotropy of all Mn complexes. The experimental spectra and simulations are shown in Fig. 2. For each HF-ESR frequency and temperature, we recorded two experimental spectra, one sweeping the magnetic field upfield and the other one downfield. Due to the large self-inductance of the superconducting magnet, an artificial hysteresis appears between these two spectra. We chose to present the raw recorded spectra, without any data treatment to correct this hysteresis. For the simulation, we aim at reproducing the medium value (between upfield and downfield) for the resonance positions. This is illustrated on the zoomed HF-ESR spectra (Fig. 2**e**) where the simulated spectrum is presented between the downfield and upfield experimental recordings. As expected, this HF-ESR study, performed on powdered samples, allowed establishing accurate values for the ZFS anisotropy (in magnitude and sign) but it is less suited to obtain very precise parameters for the hyperfine coupling. So, only an isotropic hyperfine parameter is considered. Also, the linewidth for the individual transition is kept constant for all transitions whereas the central transitions are clearly narrower than the external ones. Therefore the relative intensities are not well reproduced.

HF-ESR measurements were performed on 1% magnetic diluted powder samples , namely $[\mathrm{Zn_{0.99}Mn_{0.01}(Me_6tren}X)]^+$ [X = Cl (**1**), X = Br (**2**) and X = I (**3**)]. The spectra were recorded at 255.36 GHz for all three compounds with additional measurements conducted at 331.2 GHz for **3** owing to its stronger zero-field splitting.

The results are shown in the Fig. 2. The recorded spectra can be well described with $S = 5/2$ and $I = 5/2$. Throughout the series, the intensity of the low-temperature spectra clearly shifts from high magnetic fields, i.e. above the $g = 2$ resonance field for **1**, towards low magnetic fields, i.e. below $g = 2$, for **2** and **3**, indicating the molecular magnetic anisotropy can be tuned from easy-axis type ($D < 0$) to easy-plane type ($D > 0$) with varying the axial

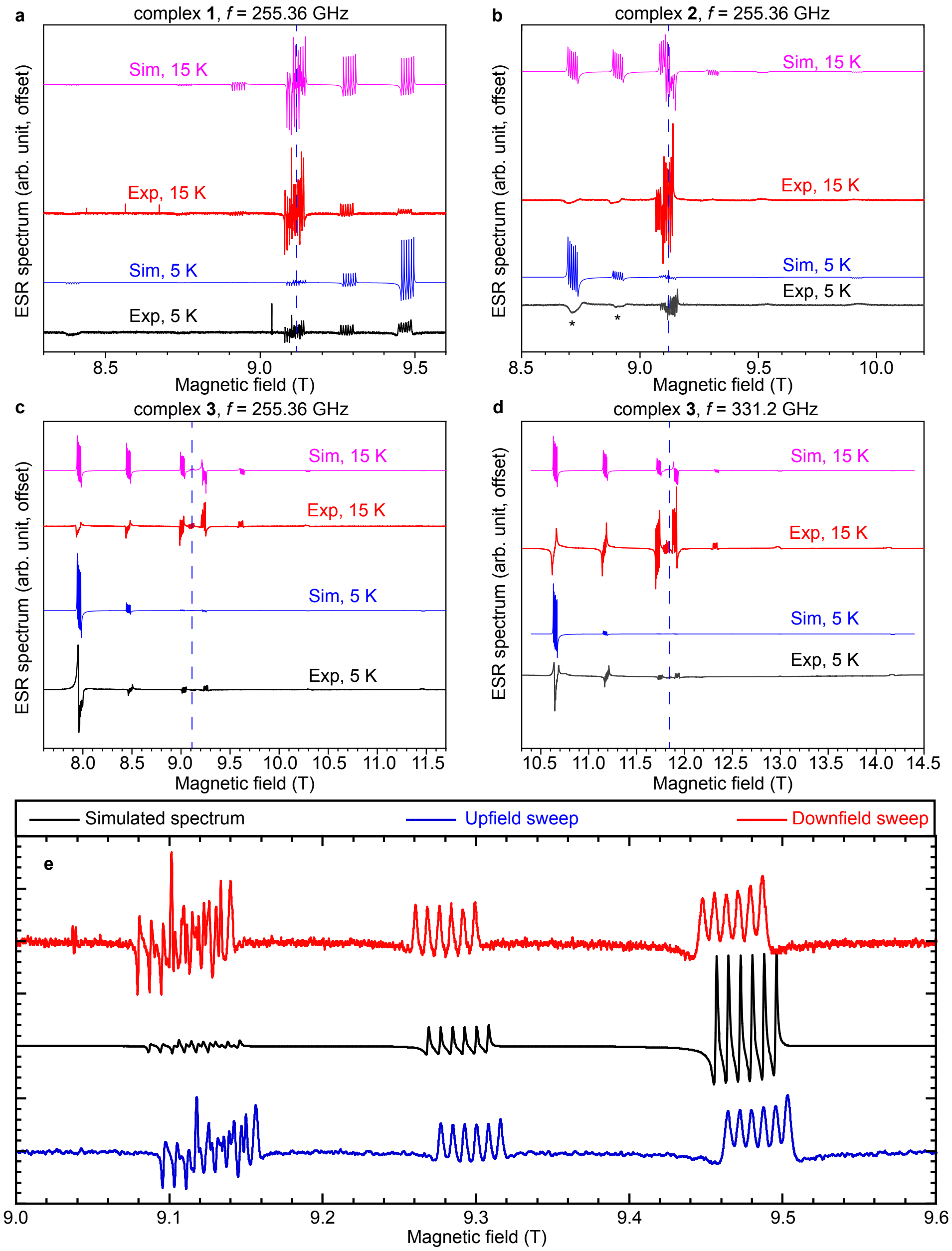

Supplementary Fig. 2. **HF-ESR spectra and simulations.** Low temperature High-frequency continuous-wave ESR measurements for complexes **1** **(a)**, **2** **(b)** and **3** [**(c)** and **(d)**]. The $g = 2$ resonance field for an isotropic spin 5/2 systems is indicated by the vertical dashed lines. **(e)**. Zoom in for the 5 K spectrum for **1** showing the hysteresis in the field sweep and the comparison between the experimental and simulated spectra.

coordination atom X. The data for **1** can be explained with an easy-axis type anisotropy of $D = -0.168$ cm^{-1}. **2** and **3** exhibit easy-plane type anisotropy with $D = +0.188$ and $+0.55$ cm^{-1}, respectively. The hyperfine coupling is almost identical in the family with $|A| = 7.3 \times 10^{-3}$ cm^{-1} for **1** and **2** and $|A| = 7.1 \times 10^{-3}$ cm^{-1} for **3**.

It is worth noting that the spectra for **2** exhibit two broad features due to unresolved hyperfine structure, as indicated by * symbols in Fig. 2**(b)**, whereas all hyperfine features are clearly resolved in the spectra for **1** and **3**. This can only be explained by including a substantial larger D-strain parameter for **2**. Increasing the overall linewidth, A-strain or g-strain in the simulation leads to unresolved hyperfine features for all transitions, which is inconsistent with the data. This is consistent with the pulsed ESR data for **2** (see below), where only the inter-Kramers transitions show unresolved hyperfine features and all the nuclear spin states can still be clearly identified for the intra-Kramers transitions.

IV. Pulsed electron spin resonance and measurements for spin-electric couplings

A. Spin coherence measurements

The spin-lattice relaxation time (T_1) is measured employing the standard inversion recovery sequence [$\pi - T - \pi/2 - \tau - \pi - \tau -$ echo] and fitting the echo intensity vs. T using a single-exponential function. In the T_1 measurements, we used a fixed $\tau = 600$ ns for all complexes and varied the delay (T) between the first π and the $\pi/2$ pulses. The phase coherence time (T_m) is measured by the standard Hahn-echo sequence [$\pi/2 - \tau - \pi - \tau -$ echo], where τ is varied in the experiments. All molecules exhibit strong electron spin echo envelop modulations (ESEEM) and decays cannot be explained by a simple monoexponential, suggesting the dephasing process is affected by several mechanisms. The data are fitted with a stretched exponential function of the form [5]:

$$Y(2\tau) = Y(0)\exp(-(2\tau/T_m)^x). \tag{1}$$

The pulsed Q-band ESR spectra for **1** and **2** are shown in the main text (and Fig. 6**a** and Fig. 7**a**). The single-crystal Q-band echo-detected field sweep (EDFS) spectra for **3**, on the other hand, are more challenging to interpret. Fig. 3**(a)** shows the representative Q-band spectrum recorded for **3**. The spectrum was recorded at 5 K with the magnetic field applied (nominally) parallel to the [111] axis of the crystal.

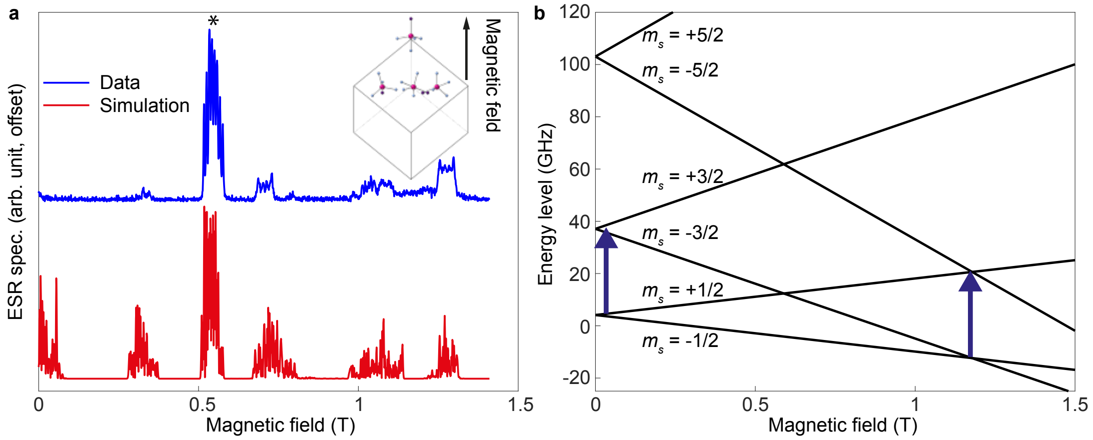

Supplementary Fig. 3. **Q-band pulsed ESR data for compound 3. (a)**. Representative EDFS spectrum for **3** recorded at 34 GHz, 5 K. The simulation is performed using the ZFS parameters in the main text with the magnetic field aligned 3.8° away from the [111] crystallography orientation, i.e. closely aligned to the Mn-I bond direction of one molecule in the unit cell, as illustrated by the top-right inset. Spin relaxation measurements were performed at 0.58 T as indicated by the * symbol. **(b)**. The energy diagram for **3** with the magnetic field parallel to its ZFS axis. The dark blue arrows indicated the observable Q-band transitions at 34 GHz. Note the width of the arrow head is chosen to represent the spread of the ESR transitions due to hyperfine interactions. As indicated by the arrow above 1 T, both the $m_s = -3/2 \leftrightarrow m_s = -5/2$ and the $m_s = -1/2 \leftrightarrow m_s = +1/2$ transitions occur at approximately the same magnetic field range for the Q-band frequency.

As described in Table 1, **3** crystallize in a cubic space group with the unit cell containing four inequivalent subspecies with non-parallel Mn-I bonds. Therefore, the local magnetic anisotropy axes for these four molecules, assuming parallel to the Mn-I bond, are not parallel to each others. Here we focus on the molecule with its Mn-I bond parallel to the [111] axis in the analysis for clarity. The conclusion can be applied to any subspecies as they are related by the symmetry operations associated with the crystallography space group. If an external magnetic field is applied parallel to the [111] axis of the crystal, this field would be parallel to the anisotropy axis of one of the four molecules within the unit cell, but almost perpendicular to (109.47° away from) the anisotropy axes of the other three molecules. In fact, it is impossible to apply a magnetic field that has the same relative orientation against the anisotropy axes of all four inequivalent molecules. Therefore, any single-crystal ESR spectrum for **3** always includes contributions from molecules with different orientation. This is particularly true for the low-field part of the spectrum, where the ZFS is much stronger than the Zeeman interactions and the ESR transitions for the four inequivalent molecules are not well-separated by the applied magnetic field.

It is possible to select the molecule with its Mn-I bond parallel the [111] axis at high magnetic fields, where its ESR transitions is separated from the other three molecules. There are two possible ESR transitions that can be accessed with the Q-band frequency above 1 T, which are the transitions between $m_s = -3/2 \leftrightarrow m_s = -5/2$ and $m_s = -1/2 \leftrightarrow m_s = +1/2$. However, the combination of the ZFS of **3** (+0.55 cm^{-1}), and the Q-band experimental

frequency (∼ 34 GHz) leads to an accidental match between the resonance condition of these two transitions that they occur at the same magnetic field range, as indicated by the blue arrow between 1 T and 1.5 T in Fig. 3**b**. This overlapping of these two different ESR transition makes it intractable to assign the resonances unambiguously.

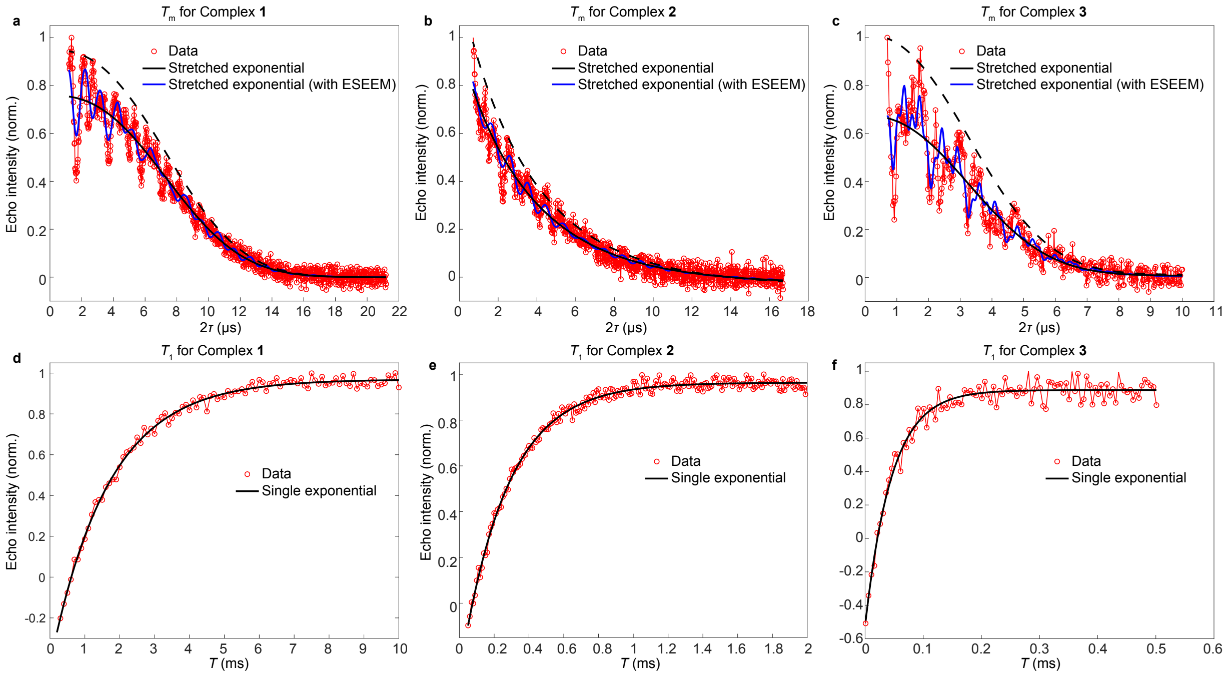

Supplementary Fig. 4. **Representative relaxation behaviour for compounds 1, 2 and 3.** The quantum phase memory time (T_m, upper panels) and spin lattice relaxation time (T_1, lower panels) are measured for all molecules. The black dashed curves in **(a)** - **(c)** are the *simulations* for the top of the ESEEM traces using the parameters in Table 4. All data were recorded at 5 K.

The representative relaxation time measurement data for all complexes are shown in Fig. 4. The relaxation times for **1** and **2** are measured with the $m_s = -3/2 \leftrightarrow m_s = -5/2$ and $m_s = +3/2 \leftrightarrow m_s = +5/2$ transitions, respectively. The relaxation time for **3** is measured at 0.58 T, as indicated by * in Fig. 3**a** for signal-to-noise reasons. All T_1 relaxation time measurements can be successfully fitted using a single exponential decay. However, we have observed noticeable ESEEM patterns for all complexes, and the relaxation behaviours cannot be explained by a simple exponential decay. Consequently, they are fitted using Equation 1.

Supplementary Table 4. **The fitted phase coherence time T_m, stretch parameters x and spin-lattice relaxation time T_1 for all complexes.**

	complex **1**			complex **2**			complex **3**		
	T_m (μs)	x	T_1 (ms)	T_m (μs)	x	T_1 (ms)	T_m (μs)	x	T_1 (ms)
3.5 K	8.79 ± 0.11	2.32 ± 0.10	2.34 ± 0.05	5.62 ± 0.12	1.65 ± 0.09	0.306 ± 0.018	4.78 ± 0.19	2.27 ± 0.30	0.080 ± 0.003
5 K	8.85 ± 0.11	2.47 ± 0.11	1.67 ± 0.05	3.61 ± 0.22	0.94 ± 0.08	0.266 ± 0.007	4.20 ± 0.18	2.31 ± 0.33	0.045 ± 0.003
7 K	8.76 ± 0.14	2.49 ± 0.14	0.78 ± 0.05	2.72 ± 0.24	0.76 ± 0.07	0.134 ± 0.004	3.66 ± 0.17	2.18 ± 0.33	0.031 ± 0.004
10 K	8.42 ± 0.16	2.49 ± 0.16	0.21 ± 0.02	1.79 ± 0.25	0.70 ± 0.07	0.051 ± 0.002	3.03 ± 0.23	2.19 ± 0.53	0.017 ± 0.002
15 K	7.33 ± 0.19	1.92 ± 0.14	0.039 ± 0.003	0.25 ± 0.28	0.39 ± 0.11	0.015 ± 0.001	2.55 ± 0.26	2.25 ± 0.74	0.010 ± 0.001
20 K	5.27 ± 0.23	1.61 ± 0.17	0.012 ± 0.002	0.29 ± 0.17	0.47 ± 0.09	0.006 ± 0.001	2.04 ± 0.47	1.57 ± 0.71	0.005 ± 0.001

The fitted results and the their uncertainties are listed in Table 4. In fitting T_m, it is possible to include decaying oscillation components to reproduce the ESEEM patterns, as shown by the blue lines in Fig. 4. However, the inclusion of the oscillation components has little effect on T_m and x. Hence, the values in Table 4 are obtained using Equation 1 only and fitting the entire ESEEM traces (black solid curves in Fig. 4**a-c**). This is further supported by simulating the top of the ESEEM envelops [6], as shown by the black dashed curves in Fig. 4**a-c**. The simulations were performed using the parameters in Table 4 and Equation 1, with the overall amplitude of the signal, $Y(0)$, as the only adjustable

parameter in the simulations. The simulations match with the top of the ESEEM envelopes well, confirming the T_m and x values in Table 4 provide a good description for the phase decoherence behaviours for all complexes.

All three complexes exhibit similar temperature dependences for the relaxation times. The T_1 for **2** is significantly shorter than that for **1**, which suggest the spin energy of **2** is more susceptible to low-energy phonon modes of the molecules. This is consistent with the significantly stronger SEC and larger D strain for **2**. We also note that the measured T_1 for **3** is the shortest among all three complexes, which is consistent with its strongest SEC. However, since it is difficult to assign the Q-band ESR transition for **3** unambiguously, one should avoid a direct quantitative comparison between the relaxation results for **3** and those for **1** and **2**.

B. Spin-electric coupling measurements

Fig. 5 shows the configuration for the SEC measurement. The E-field pluses are generated by applying voltage pulses to a pair of conductive plates that are inserted into the microwave resonators (the blue transparent plates in Fig. 5**a**). In order to achieve independent rotations of the sample and E-field orientations, we mount the sample and the E-field apparatus separately. The conductive plates are inserted to the microwave resonator from the *top* of the Q-band (for **1** and **2**) or X-band (for **3**) probe, which is typically used for sample insertion in standard ESR experiments. The plates, i.e. the E-field orientation, can be rotated in the y-z plane manually as indicated in Fig. 5**b**. A single-crystal sample is mounted inside a quartz tube with the outer/inner diameter of 1.8/1.2 mm. Numerical simulation of the E-field shows the parallel conductive plates generate a homogeneous electric field on the crystal (Fig. 5**c**). The quartz tube is then attached to a 3 mm PTFE rod and inserted into the microwave resonator from the *bottom* of the probe. The PTFE rod is mechanically fixed to the bottom bore of the resonators. To reduce the

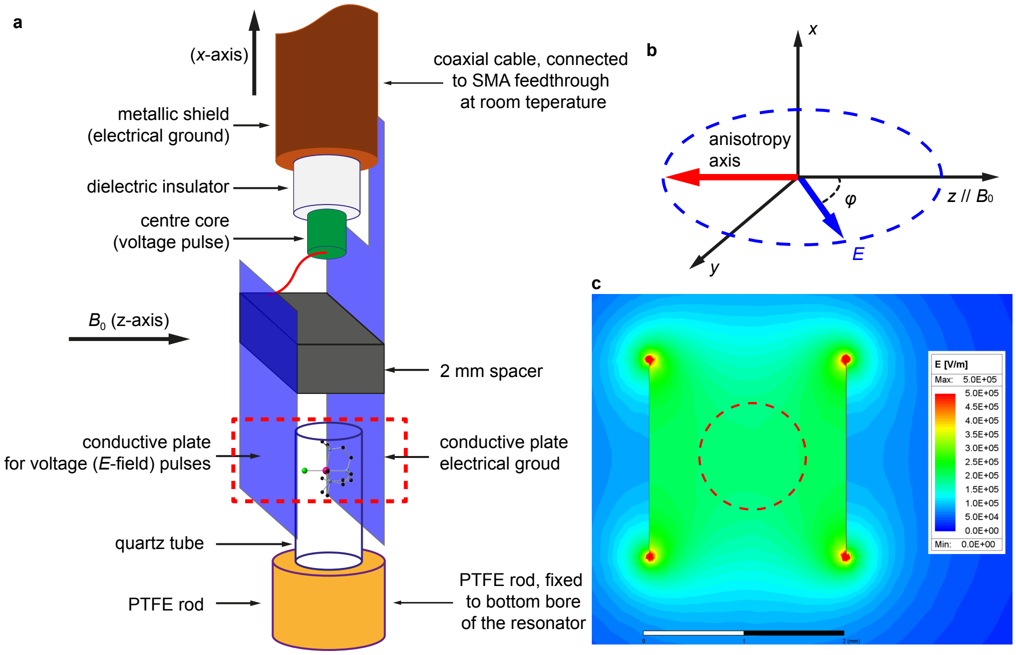

Supplementary Fig. 5. **SEC experimental setup. (a)**. Schematic showing the SEC experimental configuration. The electric field pulses were generated by applying voltage pulses to two parallel conducting plates, as indicated by the blue semi-transparent plates. One of the plates (on the right) is directly attached to the metallic shield of the coaxial cable serving as the electrical ground. The other plate (on the left) is attached to the centre core of the coaxial cable via a short piece of wire ($<$ 10 mm), as indicated by the red curve. The coaxial cable is connected a hermetic SMA feedthrough at the top of the apparatus (not shown), which is connected to the voltage pulse generator. A single crystal sample, indicated by the picture of the molecules, is mounted in a quartz tube and inserted between the plate. The red dashed box indicates the section of the apparatus inserted inside the microwave resonators **(b)** The B_0, E-field and sample orientations in the experiments. The magnetic field was applied parallel to the z-axis (in the x-y-z laboratory frame). The sample was oriented with the molecular magnetic anisotropy axis (the red arrow) parallel to B_0 (more details are provided in the following figures). The E-field orientation φ was rotated within the y-z plane. **(c)** Numerical simulation of the electric field between the plates. The red dashed circle indicates the interior region of the quartz tube where the sample is mounted. The simulation was performed with 400 V applied across the plates.

effect on the modes of the microwave resonators, only a portion of the conductive plates, the sample and the quartz tube are inserted inside the resonators, as indicated by the red dashed box in Fig. 5**a**.

The orientation of the sample can also be rotated in the y-z plane manually by rotating the entire probe against B_0. The Q-band and X-band probes are connected to the microwave bridge using a flexible WR28 waveguide (26.5 - 40 GHz) and a flexible coaxial cable with SMA connectors (DC - 18 GHz), respectively. These connections allow sufficient mechanically flexibility for rotating the entire probes. The sample and E-field can be rotated independently around the x-axis in this configuration, allowing us to orientate the sample against B_0 first. In the angle-dependence SEC measurements, the sample is fixed against B_0, and the E-field is rotated against the crystal.

The application of an electric field modulates the Hamiltonian of the molecular spin by SEC, resulting in a frequency change (δf_E) in the ESR transitions that can be deduced from the E-field dependence of the echo signal. For **1** and **2**, both inter-Kramers ($\pm5/2 \leftrightarrow \pm3/2$ and $\pm3/2 \leftrightarrow \pm1/2$) and intra-Kramers ($-1/2 \leftrightarrow +1/2$) transitions can be observed in the frequency/magnetic field range of a Q-band ($\sim$ 34 GHz) pulsed ESR spectrometer (Fig. 6 and 7), allowing us to investigate the SEC of all the parameters separately. Measuring the SEC for **3** was more challenging owing to its larger ZFS and non-axial crystal packing, making the Q-band data intractable. Therefore, the SEC for **3** is measured at X-band ($\sim$ 10 GHz) with a nominally forbidden ESR transition (see below).

The energy diagram and the representative EDFS spectrum for **1** are shown in Fig. 6**a**. Three ESR transitions were observed at the Q-band ($\sim$34 GHz) frequency, corresponding to transitions associated with different m_s projections shown in the energy diagram. The fine features in the spectrum are due to hyperfine interaction.

The existence of a unique molecular orientation in the single-crystal sample of **1** allows us to determine both

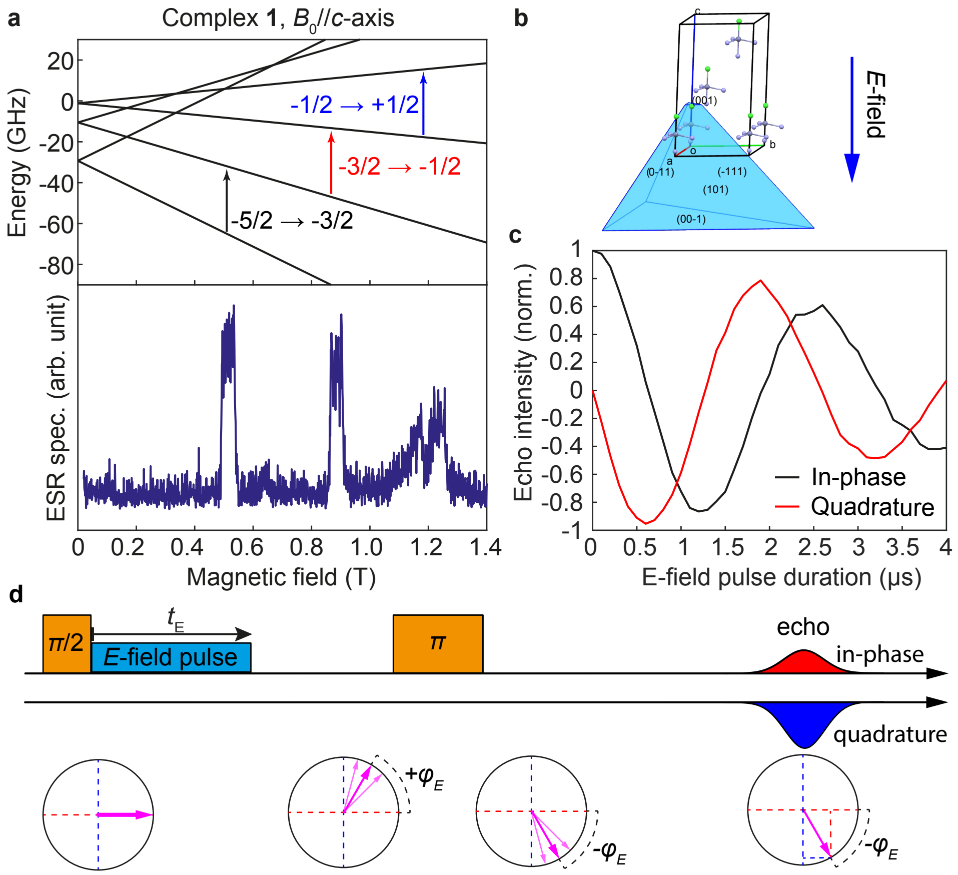

Supplementary Fig. 6. **Representative SEC data for compound 1.** **(a)** The energy diagram (top) and EDFS spectrum for **1**. **(b)** Schematic figure indicating the shape of the single crystal used for SEC measurements and the orientation of the molecules within the crystal. **(c)** Representative SEC measurement performed with the $-5/2 \rightarrow -3/2$ transition with $\tau = 4\mu$s for the microwave sequence. **(d)** The pulse sequence used for detecting SECs (top). The microwave and electric field pulses are represented by the orange and blue squares, respectively. (bottom) The spin evolution schemes (in the xy-plane of the Bloch sphere) depicting the correlation between the observed echo signal and the SEC. The red and blue dashed lines represent the x (in-phase) and y (quadrature) channels, respectively. The arrows correspond to the transverse magnetism, i.e. spin echo, of the sample. All data were recorded at 3.5 K.

the magnitude and the polarity of the SEC, as illustrated in Fig. 6**d**. For a given ESR transition, the first $\pi/2$ microwave pulse generates a coherence and rotates the spins parallel to the x-axis in the rotating frame defined by the microwave. A dc E-field pulse applied immediately after the $\pi/2$ pulse modifies the ESR transitions frequency by δf_E via the SEC, leading to an additional phase factor of $\varphi_E = \delta f_E \times t_E$ for the evolution period between the $\pi/2$ and π pulses. This phase is then inverted by the refocusing π pulse and the polarity is extrapolated from the duration (t_E) dependence of the quadrature part of the echo signal: $+\sin 2|2\pi f_E \times t_E|$ for $\delta f_E < 0$ and $-\sin|2\pi f_E \times t_E|$ for $\delta f_E > 0$. By performing X-ray crystallography measurements on the same crystals used in SEC experiments, it allows us to associate the polarity of the SEC with the molecular structure unambiguously.

Fig. 6**c** shows the echo signal of the $-5/2 \to -3/2$ transition as a function of t_E for **1**. The quadrature part of the signal follows a $-\sin t_E$ function upon the application of an E-field pulse, suggesting $\delta f_E > 0$. The transition frequency equals to $-4D + \mu_B g B$, $(D < 0)$. Since the electric field is only strongly coupled to the zero-field splitting parameter, the E-field sensitivity of the D can be calculated using $\delta D_E = -\delta f_E/4$ for **1**. An increase in the ESR transition frequency suggests the magnitude of D increases and $\delta D_E (= D(E) - D(E=0)) < 0$ for **1** when an electric field is applied in the Cl-Mn-N direction (Fig. 6**b**).

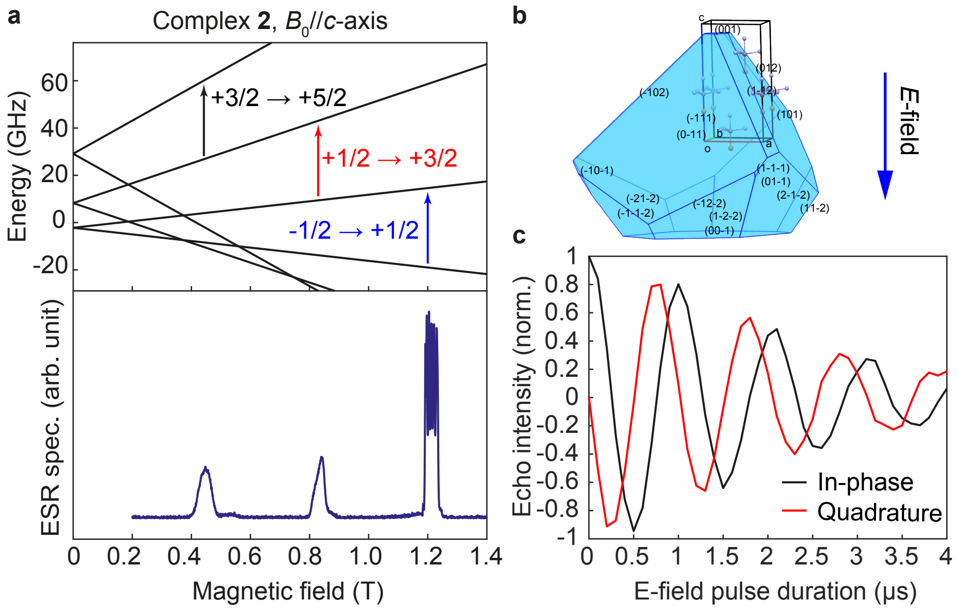

Supplementary Fig. 7. **Representative SEC data for compound 2.** **(a)** The energy diagram (top) and EDFS spectrum for **2**. Note the hyperfine features can only be resolved for the $-1/2 \to +1/2$ transition, not the other two inter-Kramers transitions. **(b)** Schematic figure indicating the shape of the single crystal used for SEC measurements and the orientation of the molecules within the crystal. **(c)** Representative SEC measurement performed with the $+3/2 \to +5/2$ transition with $\tau = 4\mu$s for the microwave sequence. All data were recorded at 3.5 K.

The polarity of the SEC for **2** is determined using the same method. Fig. 7**(c)** shows the echo signal of the $+3/2 \to +5/2$ transition as a function of t_E for **2**. A similar $-\sin t_E$ dependence was observed for the quadrature part of the echo, suggesting $\delta f_E > 0$. The transition frequency equals to $4D + \mu_B g B$ $(D > 0)$, the E-field sensitivity of the anisotropy parameter D can be calculated as $\delta D_E = \delta f_E/4$ for **2**. A positive $\delta f_E > 0$ indicates the magnitude of D increases and $\delta D_E (= D(E) - D) > 0$ in this particular E-field orientation. However, the X-ray data of the crystal shows that the electric field was applied in the N-Mn-Br direction, as shown in Fig. 7**b**. Hence, if an E-field pulsed is applied in the Br-Mn-N direction, similar to the experimental configuration in Fig. 6**b**, one should also expect $\delta D_E < 0$ for complex **2**.

Measuring the SEC for complex **3** is more challenging due to the reasons discussed in the previous section. Hence, we choose to investigate the SEC for **3** using the X-band (~9.8 GHz) frequency instead. The energy diagram and the EDFS spectrum are shown in Fig. 8**a**. The crystal was rotated with the B_0 field slightly tilted away from the C_3 axis of one molecular subpopulation. This small tilt mixes states with different m_s, thus making a nominally forbidden transition $(-3/2 \to +1/2, \delta m_s = 2)$ visible in the ESR experiment, as indicated by the red arrow in the top panel of Fig. 8**a**. Both the numerical simulations and the experimental data show that this transition is only observable within a small range of tilting angles, approximately between 7° to 13°. Outside this range, the resonance either becomes too broad (for large tilt) or the transition probability vanishes (for small tilt), making the resonance indistinguishable from the spectrum baseline. Importantly, only one molecular subpopulation will contribute to this resonance, while

X-band transitions for the other three subpopulations only appear at much lower fields, contributing to the large feature centred around 0.3 T as indicated by the blue arrows in Fig. 8**a**. Hence, this experimental configuration allows us to select one molecular subpopulation and investigate the correlation between the SEC and its structure for **3**.

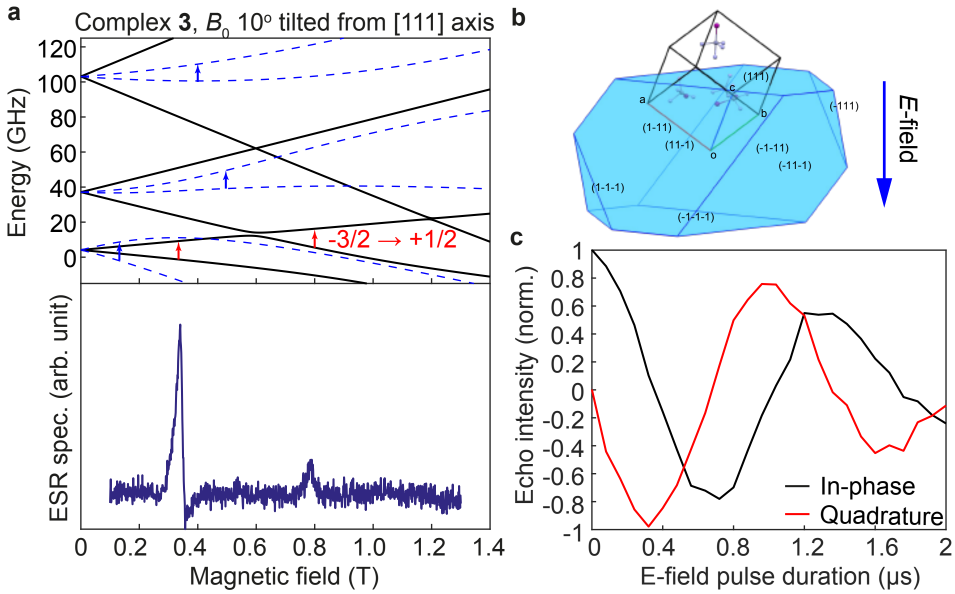

Supplementary Fig. 8. **Representative SEC data for compound 3.** **(a)** The energy diagram (top) and EDFS spectrum for **3**. The black solid lines in energy diagram correspond to the energy levels of the molecule with B_0 tilted 10° away from the magnetic anisotropy axis. The dashed blue lines (simulated with B_0 being 100° away from the magnetic anisotropy axis) illustrate the energy levels of the molecules with their magnetic anisotropy axes roughly perpendicular to B_0. The red and blue arrows indicate the possible X-band ESR transitions. **(b)** Schematic figure indicating the shape of the single crystal used for SEC measurements and the orientation of the molecules within the crystal. **(c)** Representative SEC measurement performed with the $-3/2 \rightarrow +1/2$ transition with $\tau = 2\mu$s for the microwave sequence. All data were recorded at 3.5 K.

The representative data are shown in Fig. 8**c**. The quadrature part of the echo signal follows a $-\sin t_E$ function, suggesting $\delta f_E > 0$. The frequency of the ESR transition equals to $2\mu_B gB - 2D$ for $D > 0$ (note $2\mu_B gB > 2D$ at the resonance field). The extra factor of 2 for the Zeeman term is due to $\delta m_s = 2$. Assuming only the zero-field-splitting parameter is modified the external electric field (based on the results for **1** and **2**), the E-field sensitivity of the anisotropy parameter D can be calculated as $\delta D_E = -\delta f_E/2$ for **3**. Therefore, the data indicate a $\delta D_E < 0$ when the external electric field is applied in the I-Mn-N direction.

Supplementary Table 5. **The magnetic anisotropy and the SEC for the molecules.**

	D (GHz)	Polarity of δD_E (E-field in the X-Mn-N direction)	Maximum $\lvert\delta D_E/E\rvert$ Hz/(V/m)	$\lvert\delta D_E/E\rvert/\lvert D\rvert$ 10^{-9} Hz/(V/m)
**1**	-4.7	negative	0.42	0.089
**2**	5.2	negative	1.2	0.231
**3**	16.5	negative	1.7	0.103

The results are summarised in Table 5. While the magnetic anisotropy of the molecule changes from easy-axis ($D < 0$) to easy-plane ($D > 0$) type with varying the axial halogen atom, the polarity of the SEC remains the same, that an electric field applied in the X-Mn-N direction always shifts the anisotropy towards the easy-axis type. In addition, the electric field sensitivity, $\lvert\delta D_E/E\rvert$, is also not proportional to the magnitude of D, suggesting the SEC is due to interplays between different contributions in these complexes.

V. Computational details

A. The ZFS parameter for the [Mn(me$_6$tren)X]Y_2 molecules

Ab initio calculations were performed on the three complexes, but the full analysis was carried out on the chlorine (**1**) and the iodine (**3**) derivatives to understand the trends observed experimentally. First, we study the complexes in the absence of an electric field to understand the evolution of the ZFS axial parameters D (see Table 1 in the main text) within the series. The values are very low because the spin-orbit interaction involves only states of different spin multiplicity, namely the sextuplet ground state (^6A, Mn(II), d^5, $s = 5/2$) and the quadruplet excited states. The value of D for **1** calculated using the X-Ray structure is in perfect agreement with the experimental one (determined by HF-HFEPR) while that of **3** is underestimated (0.098 vs. 0.550 cm^{-1}). The trend is, however, reproduced. It is worth noting that the same trend is observed when the geometries considered are those optimized by DFT while preserving the C_3 symmetry point group. It is therefore on these geometries that the following theoretical analysis is performed. It should be noted that calculating and rationalizing such minute values is a real challenge for theory. Despite this difficulty, the analysis we made allows rationalizing the trends observed experimentally for both the effect of the axial ligand and that of the electric field as detailed below.

To rationalize the experimental D-values trend, we compare the contributions to D of the excited states for **1** and **3** since the ZFS axial parameter of the $s = 5/2$ manifold ($m_s = \pm 5/2, \pm 3/2$ and $\pm 1/2$) is due to the interaction via the spin-orbit operator between the ground state (^6A) and the excited quadruplet states obtained from excitations between the d orbitals. The contributions of the m_{sl} components of each ith excited quadruplet state $^4Y^i$ with the excitation energy $\mathcal{E}(^4Y^i)$ from the ground state ^6A are calculated *ab initio* and extracted using the effective Hamiltonian theory implemented in the ORCA code. Nevertheless, they can also be analyzed by means of the second order perturbation theory using the following expression:

$$c(D)[^4Y^i_{m_{sl}}] = \sum_k \frac{|\langle ^6A_{m_s}|\, \zeta_k[(\hat{L}_+\hat{S}_- + \hat{L}_-\hat{S}_+)/2 + \hat{L}_z\hat{S}_z]\, |^4Y^i_{m_{sl}}\rangle|^2}{\mathcal{E}(^4Y^i)} \quad (2)$$

Supplementary Table 6. **Theoretical calculations for the magnetic anisotropy of 1 and 3.** $C(D)$ are the contributions of the most contributing quadruplet states;; the index i for the states E^i (doubly degenerate) or A^i (non-degenerate) is assigned based on the state's energetic order (from lowest to highest). $\mathcal{E}(^4Y^i)$ is the excitation energies from the ground state. $\Delta C(D)$ and $\Delta\mathcal{E}(^4Y^i)$ are the difference between **1** (Cl) and **3** (I). SOC^2 are the squared module of the SOC between the ground and excited states. The given couplings are between the $m_s = 5/2$ component of the ground state and the $m_{sl} = 3/2$ component of the excited quadruplets for the $\hat{L}_+\hat{S}_- + \hat{L}_-\hat{S}_+$ part of the SOC operator, and between the $m_s, m_{sl} = 1/2$ components of the ground and excited states for the $\hat{L}_z\hat{S}_z$ part. The variations between **1** and **3**, ΔSOC^2, are provided in the last two columns. All quantities are given in cm^{-1}.

	**1**		**3**				**1**		**3**			
$^4Y^i$	$C(D)$	$\mathcal{E}(^4Y^i)$	$C(D)$	$\mathcal{E}(^4Y^i)$	$\Delta C(D)$	$\Delta\mathcal{E}(^4Y^i)$	SOC^2 5/2 − 3/2	SOC^2 1/2 − 1/2	SOC^2 5/2 − 3/2	SOC^2 1/2 − 1/2	ΔSOC^2 5/2 − 3/2	ΔSOC^2 1/2 − 1/2
E^1	-0.672	19940	-0.544	20805	0.128	865	67108	0	56511	0	-10597	
A^1	0.307	24295	0.296	24457	-0.011	162	0	44706	0	43399		-1307
E^2	-0.090	28996	-0.044	29533	0.046	537	13065	0	6548	0	-6517	
A^2	1.291	31314	1.384	31220	0.093	-94	0	242535	0	259376		16841
E^3	-0.284	30879	-0.314	31230	-0.030	351	43870	0	48954	0	5084	
E^4	-0.674	34250	-0.612	33527	-0.062	-723	115596	0	102523	0	-13073	
$\sum C(D)$	-0.122		0.166		0.288							
D	-0.130		0.174		0.304							

This expression shows that $c(D)$ is: i) proportional to the magnitude of the spin-orbit coupling (SOC) between the ground and excited states and ii) inversely proportional to the excitation energies. By summing over the m_s and m_{sl} components of both the excited and the ground states, one gets the full contribution $C(D)$ of each excited state. Among the 24 quadruplets, we observe that only 6 are contributing quantitatively: i) 4 doubly degenerate states noted E^i ($i = 1$ to 4) which are coupled through the $\hat{L}_+\hat{S}_- + \hat{L}_-\hat{S}_+$ part of the SOC and therefore stabilizes the highest m_s components ($\pm 5/2$) of the sextuplet, generating a negative contribution to D, ii) two non-degenerate states noted A^i

($i = 1, 2$) which are coupled through the $\hat{L}_z\hat{S}_z$ part of the spin-orbit operator that stabilises the lowest m_s components of the sextuplet, hence contributing positively to D. In Table 6, we reported the values of the contributions to D of these states, together with their excitation energies and the most important SOCs (squared for direct comparison with Equation 2). To facilitate the comparison, we have also reported the variation of these quantities between the two complexes.

Table 6 shows the excitation energies are driven by the ligand field and follow the halogen spectrochemical series. The comparison between **1** and **3** shows that the excitation energies are slightly varied between the two molecules and the variation can be correlated to the difference in the ligand field strength. The energy of the molecular orbitals are plotted in Fig. 9. When the axial coordination atom is varied from Cl to I, the energy gaps Δ_1 [between d_{z^2} and d_{xz} (d_{yz})] and Δ_2 [between d_{z^2} and d_{xy} ($d_{x^2-y^2}$)] decrease, while Δ_3 [between d_{xy} ($d_{x^2-y^2}$) and d_{xz} (d_{yz})] increases. This behavior is in perfect agreement with the prediction of the ligand field theory in the halogens spectrochemical series, i.e. the ligand field is weaker in the Z direction for **3** than for **1**, inducing a stabilization of the orbitals with a Z component for the iodine derivative (**3**).

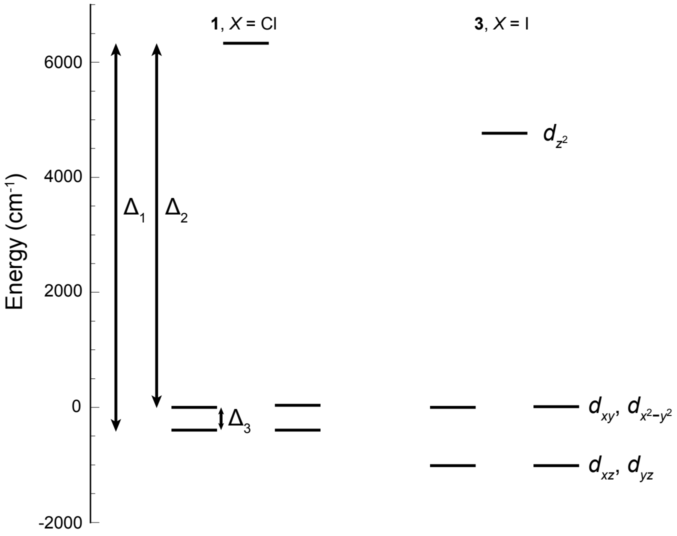

Supplementary Fig. 9. **Molecular orbitals for compounds 1 and 3.** Molecular orbitals diagram for the chlorine (left) and iodine (right) complexes in absence of E-field, the energy for the $d_{x^2-y^2}$ and d_{xy} orbitals is taken as zero of energy.

The five d orbitals are singly occupied in the sextuplet ground state while one of them is doubly occupied and another one is empty in most of the determinants of the quadruplet states considered here (see Table 7). The corresponding wave functions are reported in Table 8. It can be seen that when the main excitations from the sextuplet ground state result from a transition that promotes one electron from lower to higher orbitals, the greater the energy difference between these orbitals, the higher the energy of the state (E^1, E^2 and E^3 fall into this category). On the contrary, when excitations result from a transition from higher to lower energy orbitals, the higher the energy difference between the orbitals, the lower the state (E^3 and E^4 fall into this category). Note that E^3 contains determinants in which one electron is promoted to higher in energy orbitals and others where the electron transit from higher to lower orbitals. None of these considerations can be applied to rationalize the small variations in excitation energies of the A^i states, as excitations take place within degenerate orbitals.

While for many series of complexes, the excitation energies govern the magnitude and nature of D, the variation of the SOCs plays the most important role for the complexes investigated in this work. Indeed, one may notice that the increase or decrease of the contributions to D of an excited state is directly correlated with the decrease or increase of the SOCs (Table 6). The variation in SOCs can have two origins: either the coefficient on the $^4Y^i$ state determinants involved in the coupling varies between **1** and **3**, or the spin-orbit constants ζ_k vary. Both variations need to be considered. However, in the present case, the dominant effect concerns the spin-orbit constants. Indeed, for an excitation k involving orbital with a Z component (i.e. pointing towards the halogen), the constant ζ_k is weaker for the iodine-containing complex than for the chlorine-containing one due to the relativistic nephelauxetic effect, inducing weaker couplings and therefore lower negative contributions. The values of the mean spin-orbit constant extracted from the *ab initio* ligand field method implemented in ORCA are 310.7 cm^{-1} and 301.5 cm^{-1} for **1** and **3**, respectively. This effect prevails for the E^1, E^2 and E^4 excited states which are essentially carried by excitations involving the d_{z^2} and/or d_{xz} (d_{yz}) orbitals. Note that E^2 has a quite small SOC through the $\hat{L}_+\hat{S}_- + \hat{L}_-\hat{S}_+$ part of the spin-orbit operator, because its main coefficients are on determinants which are not coupled to the ground state

Supplementary Table 7. **Representation of the orbital occupation appearing in the sextuplet and quadruplet states.** The orbitals, from the highest to the lowest, are: d_{z^2}; d_{xy} and $d_{x^2-y^2}$; d_{xz} and d_{yz}. Note the sign of coefficients is important to fully rationalize the SOCs; as even if the weight of some configurations might be strong, their coupling to the ground state can be zero due to cancellation between different configurations.

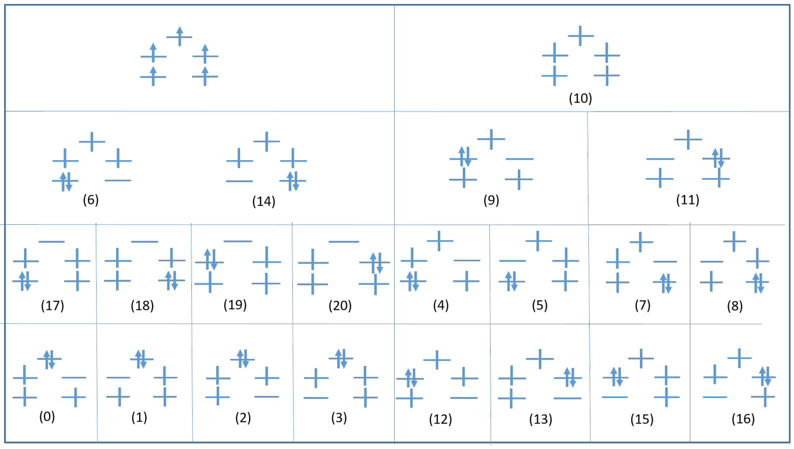

through spin-orbit excitations (here between d_{xy}, $d_{x^2-y^2}$ of $m_l = \pm 2$ and d_{z^2} of $m_l = 0$). For the E^3 state, the SOC variation is smaller and it is the weights on the main determinants [excitations from d_{xy}, $d_{x^2-y^2}$ to d_{xz}, d_{yz} (Table 7) that should be invoked. As they increase slightly, so does the SOC. Concerning the A^2 state, it is essentially carried by the two excitations from d_{xy} to $d_{x^2-y^2}$ (Table 7) and vice versa and the weight on these two configurations (Table 8) is larger for **3** than that for **1**, inducing a stronger coupling and therefore a larger positive contribution. In summary, the negative contributions to D brought by the doubly degenerate states (E^i) decrease when going from the chlorine to the iodine derivatives, while the positive contributions brought by the non-degenerate states (A^i) increases. The two effects are complementary, therefore, rationalizing the destabilization of largest $m_s(\pm 5/2)$ and the stabilization of the smallest $m_s(\pm 1/2)$, which corresponds to an increase of D when moving from the chlorine to the iodine derivative observed experimentally.

Supplementary Table 8. **Weight of the various configurations in the quadruplet states contributing to** D**.** The values are calculated for the chlorine (**1**) and iodine (**3**) complexes in zero E-field. For the E^i states, the averaged weights between the two components are presented.

	E^1		E^2		E^3		E^4		A^1		A^2	
	**1**	**3**	**1**	**3**	**1**	**3**	**1**	**3**	**1**	**3**	**1**	**3**
0, 1	.00770	.00714	.05616	.09079	.00000	.00000	.00368	.00546	.00000	.00000	.00000	.00000
2, 3	.03560	.04227	.01366	.00714	.07539	.09207	.44737	.45785	.00000	.00000	.00000	.00000
4, 5, 7, 8	.09128	.11246	.01574	.06430	.20646	.18154	.46796	.43437	.43584	.47282	.08264	.08060
6,14	.01381	.01392	.23867	.21637	.01723	.00000	.02366	.03350	.04366	.03992	.15566	.16202
9, 11	.00485	.00495	.00000	.00000	.00000	.00000	.00670	.00715	.113	.10266	.68932	.69674
10	.01866	.02218	.24176	.21739	.01961	.00251	.03036	.040655	.00000	.00000	.00000	.00000
12,13,15,16	.00000	.00000	.03408	.00000	.62654	.63636	.01216	.00770	.40164	.38464	.07129	.05854
17, 18	.77040	.74981	.00000	.00000	.05134	.07284	.00328	.00000	.00000	.00000	.00000	.00000
19, 20	0.0535	.04227	.38466	.39867	.00000	.00897	.00000	.00479	.00000	.00000	.00000	.00000

B. Spin-electric coupling for the [Mn(me$_6$tren)X]Y_2 molecules

To appreciate the effect of the electric field, we first computed the D values using the experimental structure of the two complexes for two values of the field E ($\pm 1.0284 \times 10^9$V/m). Then, we performed the same calculations for three cases: a) the DFT structure optimized without field (Table 9) as a function of the field, b) the DFT structures

optimized for each value of the field (Table 9), without applying the electric field and c) the DFT structures optimized for each value of the field as a function of the field. The results show that, although the slopes are underestimated, the trends observed experimentally are reproduced: D decreases linearly with the field applied from X^- to Mn^{2+} and the slopes are greater for **3** than for **1**, as observed experimentally. The linear behavior is maintained when considering field-distorted geometries and calculating D with (case c) or without (case b) the electric field. By comparing the theory values presented in Table 1 of the main text, one confirms that the slopes add perfectly. It is important to note that the impact of the geometrical distortion on D is larger than that of the field alone [Fig. 3 in the main text and Fig. 10 (case b)]. It is worth noting that the optimization of the structures used in the calculations was performed on a single molecule in vacuum, therefore neglecting packing and deformation effects that may be present in the crystals. This is one of the reasons for the discrepancy between calculated and experimental $\delta D/\delta E$ slopes (Table 1 in the main text).

Before analyzing the contribution to D of the different excited states in the presence of the electric field, we first examine the effect of the field on the geometrical and the electronic structures of the complexes. Table 9 gives the geometrical deformation of **1** and **3** upon the application of the electric field. One may note that the structural deformation is stronger for **3** than for **1**. The corresponding electronic effect (on **3**) can be observed by examining the energy of the molecular orbitals given by ORCA (Fig. 3 in the main text), where the energy differences Δ_1, Δ_2 and Δ_3 are increasing when the field increases. This behavior is due to the larger polarizable character of iodine, i.e. a stronger deformation of the electronic cloud induces a larger distortion of the molecule. For both complexes, the Mn-X distance increases while the Mn-N one decreases inducing a very weak destabilization of the d_{z^2} and a much weaker destabilization of the d_{xz} and d_{yz} due to the smaller π effects of the halogen for the positive field.

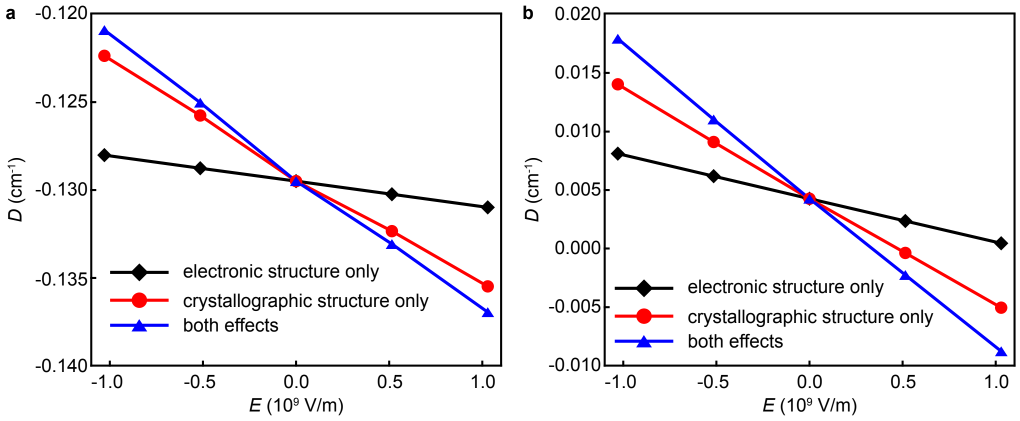

Supplementary Fig. 10. **Theoretical calculations for 1 and 2.** Theoretical calculations for **1 (a)** and **2 (b)** showing a linear SEC in both molecules. The SECs are calculated considering three configurations as described in the main text.

Supplementary Table 9. **Electric field-induced structural distortions.** Mn-X and Mn-N(apical) distances in the DFT optimized structures for **1** and **3** in the presence of an E-field field of $|E| = 1.0284 \times 10^9$V/m. $+E$ and $-E$ correspond to the field applied in the $X^- \rightarrow Mn^{2+}$ and $Mn^{2+} \rightarrow X^-$ orientations, respectively.

	**1** (X = Cl)		**3** (X = I)	
	Mn-Cl (Å)	Mn-N (Å)	Mn-I (Å)	Mn-N (Å)
$-E$	2.2684	2.3477	2.6256	2.3613
0	2.2817	2.3356	2.6446	2.3478
$+E$	2.2961	2.3300	2.6644	2.3371

In order to understand the effect of the electric field-induced deformation on the variation of the D parameters, we analyze the contribution of the excited states for **1** and **3** (Tables 10, 11 and 12). It can be seen that, when a positive E-field is applied, the positive contribution of state A^1 (containing excitations between the d_{xz} and d_{yz} orbitals) decreases (the variation of that of A^2 is almost negligible, and the negative contributions of the degenerate states E^i increase. The effect of the two types of states is complementary, and here also, the variation of the contributions follows the variation in SOC. The most affected negative contributions concern the E^3 and E^4 states. The SOC with E^3 is decreases with the field as the weight of its main configurations is decreases. On the contrary, the weight of

Supplementary Table 10. **Theoretical calculation for the SEC of compound 1.** $C(D)$ of the most contributing quadruplet states in the presence of an E-field of $|E| = 1.0284 \times 10^9$V/m applied in the positive and negative orientations.

	$-E$		$+E$				$-E$		$+E$			
$^4Y^i$	$C(D)$	$\mathcal{E}(^4Y^i)$	$C(D)$	$\mathcal{E}(^4Y^i)$	$\Delta C(D)$	$\Delta\mathcal{E}(^4Y^i)$	SOC^2 $5/2-3/2$	SOC^2 $1/2-1/2$	SOC^2 $5/2-3/2$	SOC^2 $1/2-1/2$	ΔSOC^2 $5/2-3/2$	ΔSOC^2 $1/2-1/2$
E^1	-0.659	20077	-0.684	19847	-0.026	-230	66158		67919		1761	
A^1	0.317	24226	0.297	24358	-0.020	132		46068		43377		-2691
E^2	-0.107	29003	-0.076	29014	0.032	11	15641		10892		-4749	
A^2	1.282	31370	1.298	31260	0.015	-110		241504		243531		2027
E^3	-0.296	30833	-0.27	30920	0.026	87	45637		41840		-3797	
E^4	-0.654	34195	-0.696	34280	-0.042	85	111857		119349		7492	
$\sum C(D)$	-0.117		-0.131		-0.015							
D	-0.121		-0.137		-0.016							

Supplementary Table 11. **Theoretical calculation for the SEC of compound 3.** $C(D)$ of the most contributing quadruplet states in the presence of an E-field of $|E| = 1.0284 \times 10^9$V/m applied in the positive and negative orientations.

	$-E$		$+E$				$-E$		$+E$			
$^4Y^i$	$C(D)$	$\mathcal{E}(^4Y^i)$	$C(D)$	$\mathcal{E}(^4Y^i)$	$\Delta C(D)$	$\Delta\mathcal{E}(^4Y^i)$	SOC^2 $5/2-3/2$	SOC^2 $1/2-1/2$	SOC^2 $5/2-3/2$	SOC^2 $1/2-1/2$	ΔSOC^2 $5/2-3/2$	ΔSOC^2 $1/2-1/2$
E^1	-0.524	20995	-0.562	20629	-0.038	-366	54989		57946		2957	
A^1	0.309	24403	0.283	24500	-0.026	97		45309		41546		-3763
E^2	-0.062	29566	-0.034	29515	0.028	-51	9191		4983		-4208	
A^2	1.381	31289	1.388	31152	0.007	-137		259292		259441		149
E^3	-0.348	31225	-0.27	31201	0.078	-24	54391		42244		-12147	
E^4	-0.564	33404	-0.66	33651	-0.096	247	94268		110983		16715	
$\sum C(D)$	0.192		0.145		-0.047							
D	0.199		0.149		-0.050							

the configuration in which the electrons are promoted from d_{xz}, d_{yz} to d_{z^2} increases with the field, rationalizing the increase of the SOC between the ground state and E^4.

One may wonder why the excitations among the d_{xz}, d_{yz}, $d_{x^2-y^2}$, d_{xy} orbitals, which are of symmetry E in the C_3 point group, and the d_{z^2} orbital, which is of symmetry A_1, are affected by the field along z (also of symmetry A^1). Indeed, according to group theory, $E \times A_1 \times A_1 = E$ does not contain the totally symmetric irreducible representation, so no effect should be observed. Here, ligand field theory must be invoked. The averaged orbitals in which the calculations are performed show that the essentially d_{z^2} orbital loses weight on the p_z of the halogen and gains weight on the p_z of the apical nitrogen atom as the field increases (Table 13) and is therefore not identical in the two compared calculations, rationalizing the change of weights of these configurations. The impact of the electric field is weaker for the chlorine derivative (**1**) than for the iodine one (**3**). Although the couplings are greater overall for **1** due to the weaker nephelauxetic effect, the variations with the field are much smaller. This is because Cl^- is less polarizable than I^-, inducing weaker distortions and hence a smaller variation of the coefficients. The dipole moment (Table 14) is larger in the iodine complex and its variation with field is greater, in agreement with the more significant changes in geometry under the effect of the field.

For comparison, we also present the calculation for the E-field-induced effect on the electronic structure only (Case (a) in the main text) for **1** and**3**. The results are shown in Table 15 and 16. The results follow the same trend as the ones obtained for varying the electronic structure and geometry, i.e. the variation of the contributions to D follows the variations of the SOCs.

In conclusion, *ab initio* calculations were performed to rationalize the trends observed for the axial ZFS parameter D upon changing the chemical nature of the axial ligand and varying the electric field. As the ground state of Mn(II)

Supplementary Table 12. **Weight of the various configurations in the quadruplet states contributing to D for compound 3.** The values are calculated with the two orientations of the E-field ($|E| = 1.0284 \times 10^9$V/m). For the E^i states, the averaged weights between the two components are presented.

	E^1		E^2		E^3		E^4		A^1		A^2	
	$-E$	$+E$	$-E$	$+E$	$-E$	$+E$	$-E$	$+E$	$-E$	$+E$	$-E$	$+E$
0, 1	.00667	.00640	.09434	.08768	.00000	.00410	.00437	.006010	.00000	.00000	.00000	.00000
2, 3	.04379	.04132	.01351	.00691	.10863	.07377	.43983	.47579	.00000	.00000	.00000	.00000
4, 5, 7, 8	.11158	.11080	.05090	.07890	.16002	.20684	.46180	.40128	.46364	.48232	.08208	.07916
6.,14	.01290	.01256	.21200	.21567	.00298	.00000	.03276	.03399	.04148	.03838	.16050	.16346
9, 11	.00717	.00604	.00000	.00000	.00000	.00000	.00646	.00725	.10641	.09894	.69302	.70046
10	.02538	.01860	.21337	.21738	.00797	.00175	.03971	.04128	.00000	.00000	.00000	.00000
12,13,15,16	.00000	.00000	.00000	.00000	.63822	.62182	.00000	.02500	.38846	.37556	.06196	.05520
17, 18	.73737	.76006	.00000	.00000	.07393	.07195	.00297	.00000	.00000	.00000	.00000	.00000
19, 20	.04721	.03788	.40621	.38956	.00000	.00000	.00437	.00537	.00000	.00000	.00000	.00000

Supplementary Table 13. **Coefficients of the essentially d_{z^2} molecular orbital with an external electric field of $|E| = 1.0284 \times 10^9$ V/m.**

	d_{z^2}(Mn)	p_z(I)	p_z(I)
$-E$	93.6	1.4	0.9
$+E$	93.7	1.3	1.0

Supplementary Table 14. **Electric dipole moments vs. the applied electric field.** The Electric dipole moments (μ_e) for compounds **1**, **2** and **3** are calculated as functions of the field with all three cases [$X^{-\delta}$–Mn$^{+\delta}$–N: $+E(+ \rightarrow -)$ from X to Mn].

	Case (a)			Case (b)			Case (c)		
Complex	$-E$	$+E$	$\Delta\mu_e$ (Debye)	$-E$	$+E$	$\Delta\mu_e$ (Debye)	$-E$	$+E$	$\Delta\mu_e$ (Debye)
**1**	6.886	8.702	1.816	7.660	7.907	0.247	6.755	8.818	2.063
**2**	8.900	10.826	1.926	9.729	9.996	0.267	8.764	10.958	2.194
**3**	10.864	12.988	2.124	11.767	12.103	0.336	10.700	13.165	2.465

complexes is a sextuplet, all excited quadruplet states are very high in energy. D is, therefore, very small and changes in excitation energy are not the main quantities for rationalizing variations in anisotropy. Instead, it is the SOCs that enable us to understand the observed trends. We showed that the relativistic nephelauxetic effect induces a smaller spin-orbit constant and therefore smaller SOCs in the iodine complex than in the chlorine one. As a consequence, most of the negative contributions of excited states decrease, rationalizing the change of sign of D from negative in the chlorine complex to positive in the iodine one. On the contrary, the impact of the electric field when varying from negative to positive increases the negative contributions of the excited degenerate states E^i. A separate analysis of field effects on fixed and field-distorted geometries shows that field-induced distortions play the most prominent role. Within a single complex, the changes in coefficients of the various excited states wave functions affect the SOCs and therefore the contributions to the D parameter. Comparing complexes with different halogens, we can conclude that the effects are more spectacular with iodine, which is more polarizable and for which the dipole moment is greater. Indeed, structural deformations under the effect of the field are more significant, inducing a greater variation in wave functions and therefore in SOCs.

Supplementary Table 15. **SEC for 1 calculated at the X-Ray structure.** $C(D)$ of the most contributing quadruplet states in the presence of an E-field of $|E| = 1.0284 \times 10^9$V/m applied in the positive and negative orientations.

	$-E$		$+E$				$-E$		$+E$			
${}^4Y^i$	$C(D)$	$\mathcal{E}({}^4Y^i)$	$C(D)$	$\mathcal{E}({}^4Y^i)$	$\Delta C(D)$	$\Delta\mathcal{E}({}^4Y^i)$	SOC^2 5/2 − 3/2	SOC^2 1/2 − 1/2	SOC^2 5/2 − 3/2	SOC^2 1/2 − 1/2	ΔSOC^2 5/2 − 3/2	ΔSOC^2 1/2 − 1/2
E^1	-0.738	19172	-0.744	19115	-0.006	-57	70700		71191		492	
A^1	0.291	24315	0.278	24333	-0.013	18		28266		27131		-1135
E^2	-0.060	28807	-0.052	28843	0.008	36	8639		7504		-1134	
A^2	1.294	31227	1.309	31131	0.015	-96		161638		163049		1411
E^3	-0.204	30843	-0.192	30859	0.012	16	31405		29579		-1827	
E^4	-0.748	34619	-0.762	34626	-0.014	7	129361		132076		2714	
$\sum C(D)$	-0.165		-0.163		0.002*							
D	-0.1712		-0.1749		-0.0036							

*These contributions obtained at the second order of perturbations are less precise than the values of D provided by the effective Hamiltonian theory.

Supplementary Table 16. **SEC for 3 calculated at the X-Ray structure.** $C(D)$ of the most contributing quadruplet states in the presence of an E-field of $|E| = 1.0284 \times 10^9$V/m applied in the positive and negative orientations.

	$-E$		$+E$				$-E$		$+E$			
${}^4Y^i$	$C(D)$	$\mathcal{E}({}^4Y^i)$	$C(D)$	$\mathcal{E}({}^4Y^i)$	$\Delta C(D)$	$\Delta\mathcal{E}({}^4Y^i)$	SOC^2 5/2 − 3/2	SOC^2 1/2 − 1/2	SOC^2 5/2 − 3/2	SOC^2 1/2 − 1/2	ΔSOC^2 5/2 − 3/2	ΔSOC^2 1/2 − 1/2
E^1	-0.638	19439	-0.648	19400	-0.01	-39	62087		62818		731	
A^1	0.264	24390	0.247	24471	-0.017	81		25781		24143		-1638
E^2	-0.026	29080	-0.023	29119	0.003	39	3735		3334		-401	
A^2	1.385	25447	1.399	25429	0.014	-17.7		171471		172615		1145
E^3	-0.102	30724	-0.084	30686	0.018	-38	15593		12936		-2656	
E^4	-0.782	34354	-0.802	34397	-0.02	43	134246		137984		3739	
$\sum C(D)$	0.101		0.089		-0.012							
D	0.1017		0.0876		-0.014							

[1] K. Naveen, H. Ji, T. S. Kim, D. Kim, and D.-H. Cho, C3-symmetric zinc complexes as sustainable catalysts for transforming carbon dioxide into mono- and multi-cyclic carbonates, Applied Catalysis B: Environmental **280**, 119395 (2021).
[2] G. M. Sheldrick, SHELXS-97, Program for Crystal Structure Solution, University of Göttingen, Göttingen, Germany, 1997.
[3] G. M. Sheldrick, A short history of SHELX, Acta Crystallographica Section A Foundations of Crystallography **64**, 112 (2008).
[4] L. J. Farrugia, *WinGX* suite for small-molecule single-crystal crystallography, Journal of Applied Crystallography **32**, 837 (1999).
[5] C. J. Wedge, G. A. Timco, E. T. Spielberg, R. E. George, F. Tuna, S. Rigby, E. J. L. McInnes, R. E. P. Winpenny, S. J. Blundell, and A. Ardavan, Chemical Engineering of Molecular Qubits, Physical Review Letters **108**, 107204 (2012).
[6] A. Schweiger and G. Jeschke, *Principles of Pulse Electron Paramagnetic Resonance* (Oxford University PressOxford, 2001).
