# Supplementary material for: Chemical tuning of quantum spin–electric coupling in molecular magnets
Source: Nat Chem. 2025 Aug 27;17(12):1903–9. doi: 10.1038/s41557-025-01926-5 (PMC12669029; doi:10.1038/s41557-025-01926-5)
Supplement: Supplementary file 2 — The atomic coordinates of all the optimized models used in this work. [file 41557_2025_1926_MOESM2_ESM.pdf]

Compound **1**, [Mn(me$_6$tren)Cl], geometry optimization without electric field

Mn	0.000000	0.000000	-0.487033
Cl	0.000000	0.000000	-2.769182
N	0.000000	0.000000	1.850855
N	-0.080125	2.228506	-0.037035
N	1.970005	-1.044862	-0.037035
N	-1.889880	-1.183643	-0.037035
C	-0.337747	1.366373	2.280711
C	0.281444	2.419700	1.374610
C	-1.454302	2.673528	-0.305658
C	0.834482	2.978224	-0.911674
C	-1.014440	-0.975684	2.280711
C	-2.236243	-0.966113	1.374610
C	-1.588192	-2.596226	-0.305658
C	-2.996459	-0.766429	-0.911674
C	1.352187	-0.390689	2.280711
C	1.954800	-1.453587	1.374610
C	3.042494	-0.077302	-0.305658
C	2.161977	-2.211795	-0.911674
H	-1.326652	-0.788804	3.315450
H	-0.559417	-1.966584	2.273618
H	-2.743583	-0.001229	1.442907
H	-2.953606	-1.719185	1.724891
H	-2.467525	-3.228267	-0.133553
H	-0.783710	-2.950468	0.339842
H	-1.273269	-2.705268	-1.344156
H	-2.696480	-0.882529	-1.952453
H	1.370874	-2.941369	-0.738992
H	-3.896290	-1.363426	-0.722306
H	-1.423403	1.467761	2.273618
H	-2.163325	2.153947	0.339842
H	-1.706197	2.455317	-1.344156
H	-3.232737	0.283473	-0.738992
H	1.346450	-0.754512	3.315450
H	1.372856	-2.375398	1.442907
H	2.965661	-1.698306	1.724891
H	4.029524	-0.522806	-0.133553
H	2.112533	-1.893956	-1.952453
H	3.128906	-2.692573	-0.722306
H	-0.019799	1.543316	3.315450
H	1.982820	0.498822	2.273618
H	1.370727	2.376627	1.442907
H	-0.012055	3.417491	1.724891
H	-1.561999	3.751073	-0.133553
H	2.947036	0.796521	0.339842
H	2.979465	0.249951	-1.344156
H	0.583947	2.776485	-1.952453
H	1.861864	2.657896	-0.738992
H	0.767384	4.055999	-0.722306

Compound **1**, [Mn(me$_6$tren)Cl], geometry optimization with the largest negative electric field

Mn	0.000000	-0.000000	-0.496508
Cl	0.000000	-0.000000	-2.764940
N	0.000000	-0.000000	1.851176
N	-0.078391	2.230695	-0.035826
N	1.971034	-1.047459	-0.035826
N	-1.892643	-1.183236	-0.035826
C	-0.332023	1.365450	2.278852
C	0.280970	2.419282	1.373998
C	-1.454564	2.673462	-0.301975
C	0.836982	2.984468	-0.908848
C	-1.016503	-0.970266	2.278852
C	-2.235645	-0.966314	1.373998
C	-1.588004	-2.596420	-0.301975
C	-3.003116	-0.767387	-0.908848
C	1.348526	-0.395185	2.278852
C	1.954675	-1.452968	1.373998
C	3.042568	-0.077042	-0.301975
C	2.166134	-2.217081	-0.908848
H	-1.333176	-0.776348	3.317331
H	-0.561337	-1.964747	2.281398
H	-2.747790	-0.001694	1.445401
H	-2.951311	-1.722920	1.730007
H	-2.465277	-3.229471	-0.124245
H	-0.780711	-2.945382	0.344450
H	-1.275408	-2.708104	-1.338201
H	-2.705734	-0.882464	-1.946985
H	1.376284	-2.945759	-0.732711
H	-3.899489	-1.366352	-0.715199
H	-1.420852	1.468506	2.281398
H	-2.160420	2.148806	0.344450
H	-1.707583	2.458587	-1.338201
H	-3.239244	0.280982	-0.732711
H	1.338925	-0.766391	3.317331
H	1.375362	-2.378809	1.445401
H	2.967748	-1.694450	1.730007
H	4.029442	-0.520257	-0.124245
H	2.117103	-1.902002	-1.946985
H	3.133040	-2.693881	-0.715199
H	-0.005749	1.542738	3.317331
H	1.982189	0.496241	2.281398
H	1.372428	2.380503	1.445401
H	-0.016437	3.417371	1.730007
H	-1.564165	3.749728	-0.124245
H	2.941131	0.796576	0.344450
H	2.982991	0.249516	-1.338201
H	0.588631	2.784466	-1.946985
H	1.862960	2.664776	-0.732711
H	0.766449	4.060233	-0.715199

Compound **1**, [Mn(me$_6$tren)Cl], geometry optimization with the largest Positive electric field

Mn	-0.000000	0.000000	-0.477981
Cl	-0.000000	0.000000	-2.774105
N	-0.000000	0.000000	1.851997
N	-0.082378	2.225991	-0.038275
N	1.968954	-1.041654	-0.038275
N	-1.886575	-1.184337	-0.038275
C	-0.342775	1.367664	2.283223
C	0.282717	2.419723	1.374890
C	-1.454606	2.673699	-0.308228
C	0.830983	2.971377	-0.915244
C	-1.013045	-0.980684	2.283223
C	-2.236900	-0.965022	1.374890
C	-1.588188	-2.596575	-0.308228
C	-2.988779	-0.766036	-0.915244
C	1.355819	-0.386980	2.283223
C	1.954184	-1.454701	1.374890
C	3.042794	-0.077123	-0.308228
C	2.157797	-2.205341	-0.915244
H	-1.321759	-0.800670	3.313895
H	-0.558613	-1.968175	2.267041
H	-2.738823	0.000500	1.439243
H	-2.956773	-1.713835	1.719373
H	-2.469674	-3.227303	-0.141176
H	-0.786488	-2.955732	0.336478
H	-1.271097	-2.703971	-1.348953
H	-2.685724	-0.883594	-1.958496
H	1.365343	-2.935879	-0.746615
H	-3.892183	-1.360984	-0.730442
H	-1.425184	1.467860	2.267041
H	-2.166495	2.158985	0.336478
H	-1.706159	2.452787	-1.348953
H	-3.225217	0.285518	-0.746615
H	1.354281	-0.744342	3.313895
H	1.368978	-2.372140	1.439243
H	2.962611	-1.703723	1.719373
H	4.029764	-0.525149	-0.141176
H	2.108077	-1.884108	-1.958496
H	3.124738	-2.690237	-0.730442
H	-0.032521	1.545012	3.313895
H	1.983796	0.500315	2.267041
H	1.369845	2.371640	1.439243
H	-0.005838	3.417558	1.719373
H	-1.560090	3.752452	-0.141176
H	2.952983	0.796747	0.336478
H	2.977256	0.251183	-1.348953
H	0.577647	2.767702	-1.958496
H	1.859874	2.650361	-0.746615
H	0.767444	4.051221	-0.730442

Compound **2**, [Mn(me$_6$tren)Br], geometry optimization without electric field

Mn	0.000000	0.000000	-0.122719
Br	0.000000	0.000000	-2.557526
N	0.000000	0.000000	2.217748
N	-0.075798	2.232177	0.334333
N	1.971021	-1.050445	0.334333
N	-1.895223	-1.181732	0.334333
C	-0.338997	1.365180	2.650130
C	0.282681	2.418978	1.747777
C	-1.451476	2.675402	0.067336
C	0.837505	2.998210	-0.528175
C	-1.012782	-0.976171	2.650130
C	-2.236237	-0.964681	1.747777
C	-1.591228	-2.594716	0.067336
C	-3.015279	-0.773805	-0.528175
C	1.351780	-0.389010	2.650130
C	1.953556	-1.454298	1.747777
C	3.042704	-0.080686	0.067336
C	2.177774	-2.224406	-0.528175
H	-1.321701	-0.789513	3.685889
H	-0.558189	-1.967159	2.641157
H	-2.743129	0.000273	1.818156
H	-2.953234	-1.717734	2.098734
H	-2.466791	-3.228739	0.250589
H	-0.779071	-2.943922	0.705925
H	-1.286151	-2.706300	-0.973816
H	-2.735083	-0.907217	-1.572100
H	1.380582	-2.950235	-0.367993
H	-3.912842	-1.365500	-0.313288
H	-1.424515	1.466985	2.641157
H	-2.159976	2.146656	0.705925
H	-1.700649	2.466990	-0.973816
H	-3.245269	0.279498	-0.367993
H	1.344589	-0.749870	3.685889
H	1.371328	-2.375756	1.818156
H	2.964218	-1.698709	2.098734
H	4.029565	-0.521934	0.250589
H	2.153215	-1.915043	-1.572100
H	3.138979	-2.705870	-0.313288
H	-0.022887	1.539383	3.685889
H	1.982704	0.500173	2.641157
H	1.371801	2.375483	1.818156
H	-0.010984	3.416443	2.098734
H	-1.562774	3.750673	0.250589
H	2.939047	0.797266	0.705925
H	2.986801	0.239311	-0.973816
H	0.581868	2.822260	-1.572100
H	1.864687	2.670736	-0.367993
H	0.773863	4.071371	-0.313288

Compound **2**, [Mn(me$_6$tren)Br], geometry optimization with the largest negative electric field

Mn	0.000000	0.000000	-0.132845
Br	0.000000	0.000000	-2.552647
N	0.000000	0.000000	2.216565
N	-0.073979	2.235021	0.334713
N	1.972575	-1.053443	0.334713
N	-1.898596	-1.181578	0.334713
C	-0.332695	1.364182	2.646886
C	0.281978	2.418674	1.746215
C	-1.451381	2.675907	0.070064
C	0.839834	3.005424	-0.525558
C	-1.015069	-0.970213	2.646886
C	-2.235622	-0.965137	1.746215
C	-1.591713	-2.594886	0.070064
C	-3.022691	-0.775395	-0.525558
C	1.347763	-0.393969	2.646886
C	1.953644	-1.453537	1.746215
C	3.043094	-0.081021	0.070064
C	2.182857	-2.230030	-0.525558
H	-1.328595	-0.775944	3.686758
H	-0.560233	-1.965254	2.648175
H	-2.748092	-0.000363	1.819852
H	-2.950762	-1.722299	2.103529
H	-2.465567	-3.230211	0.258651
H	-0.776664	-2.939354	0.709749
H	-1.288473	-2.709284	-0.969074
H	-2.745515	-0.907879	-1.567366
H	1.386768	-2.955487	-0.362148
H	-3.917008	-1.369346	-0.306055
H	-1.421844	1.467803	2.648175
H	-2.157223	2.142287	0.709749
H	-1.702072	2.470492	-0.969074
H	-3.252911	0.276767	-0.362148
H	1.336285	-0.762624	3.686758
H	1.374361	-2.379736	1.819852
H	2.966935	-1.694285	2.103529
H	4.030229	-0.520138	0.258651
H	2.159004	-1.923746	-1.567366
H	3.144392	-2.707556	-0.306055
H	-0.007690	1.538569	3.686758
H	1.982076	0.497451	2.648175
H	1.373731	2.380099	1.819852
H	-0.016174	3.416584	2.103529
H	-1.564662	3.750349	0.258651
H	2.933887	0.797066	0.709749
H	2.990545	0.238792	-0.969074
H	0.586511	2.831625	-1.567366
H	1.866143	2.678720	-0.362148
H	0.772616	4.076901	-0.306055

Compound **2**, [Mn(me$_6$tren)Br], geometry optimization with the largest Positive electric field

Mn	0.000000	-0.000000	-0.112951
Br	0.000000	-0.000000	-2.562683
N	0.000000	-0.000000	2.220276
N	-0.077868	2.228960	0.333912
N	1.969270	-1.047045	0.333912
N	-1.891402	-1.181916	0.333912
C	-0.344588	1.366540	2.653980
C	0.284264	2.418916	1.749053
C	-1.451967	2.674584	0.065553
C	0.834387	2.990566	-0.531437
C	-1.011164	-0.981692	2.653980
C	-2.236975	-0.963278	1.749053
C	-1.590274	-2.594732	0.065553
C	-3.007099	-0.772682	-0.531437
C	1.355752	-0.384848	2.653980
C	1.952711	-1.455638	1.749053
C	3.042241	-0.079852	0.065553
C	2.172712	-2.217883	-0.531437
H	-1.316302	-0.802466	3.685358
H	-0.557325	-1.968849	2.635195
H	-2.737736	0.002200	1.815455
H	-2.956574	-1.711643	2.093419
H	-2.467522	-3.227348	0.243845
H	-0.780932	-2.948171	0.703214
H	-1.283285	-2.704206	-0.977532
H	-2.723433	-0.907254	-1.577336
H	1.374515	-2.944309	-0.374953
H	-3.907864	-1.362288	-0.321454
H	-1.426411	1.467081	2.635195
H	-2.162725	2.150392	0.703214
H	-1.700269	2.463461	-0.977532
H	-3.237104	0.281789	-0.374953
H	1.353107	-0.738718	3.685358
H	1.366963	-2.372049	1.815455
H	2.960614	-1.704647	2.093419
H	4.028727	-0.523263	0.243845
H	2.147422	-1.904935	-1.577336
H	3.133708	-2.703165	-0.321454
H	-0.036805	1.541184	3.685358
H	1.983735	0.501767	2.635195
H	1.370773	2.369849	1.815455
H	-0.004040	3.416290	2.093419
H	-1.561205	3.750611	0.243845
H	2.943656	0.797779	0.703214
H	2.983554	0.240745	-0.977532
H	0.576011	2.812190	-1.577336
H	1.862589	2.662520	-0.374953
H	0.774156	4.065453	-0.321454

Compound **3**, [Mn(me$_6$tren)I], geometry optimization without electric field

Mn	0.000000	0.000000	0.223111
I	0.000000	0.000000	-2.421520
N	0.000000	0.000000	2.570906
N	1.968321	-1.068954	0.696015
N	-0.058419	2.239093	0.696015
N	-1.909902	-1.170139	0.696015
C	0.264114	1.380011	3.007406
C	1.063068	-0.918735	3.007406
C	-1.327182	-0.461276	3.007406
C	2.287592	-0.835582	2.112344
C	-0.420161	2.398904	2.112344
C	-1.867431	-1.563322	2.112344
C	1.288750	2.763973	0.428932
C	1.749296	-2.498077	0.428932
C	-3.038046	-0.265896	0.428932
C	3.078273	-0.601522	-0.149869
C	-1.018203	2.966623	-0.149869
C	-2.060070	-2.365101	-0.149869
H	1.341955	1.543142	2.993841
H	0.665423	-1.933738	2.993841
H	-2.007378	0.390596	2.993841
H	-0.056634	1.531782	4.045315
H	1.354879	-0.716844	4.045315
H	-1.298245	-0.814938	4.045315
H	2.740306	0.155558	2.187779
H	-1.504870	2.295396	2.187779
H	-1.235436	-2.450954	2.187779
H	3.043928	-1.548364	2.464694
H	-0.181041	3.410301	2.464694
H	-2.862887	-1.861936	2.464694
H	1.338667	3.841324	0.626240
H	2.657350	-3.079981	0.626240
H	-3.996017	-0.761342	0.626240
H	1.545012	2.584128	-0.615764
H	1.465414	-2.630084	-0.615764
H	-3.010427	0.045956	-0.615764
H	2.029843	2.268561	1.057304
H	0.949710	-2.892176	1.057304
H	-2.979553	0.623615	1.057304
H	3.235348	0.467091	-0.003436
H	-1.213161	-3.035439	-0.003436
H	-2.022186	2.568348	-0.003436
H	-0.749858	2.837163	-1.197146
H	2.831985	-0.769185	-1.197146
H	-2.082126	-2.067978	-1.197146
H	4.006841	-1.130571	0.094528
H	-1.024318	4.035311	0.094528
H	-2.982523	-2.904741	0.094528

Compound **3**, [Mn(me$_6$tren)I], geometry optimization with the largest negative electric field

Mn	0.000000	0.000000	-0.208365
I	-0.000001	-0.000002	2.417279
N	0.000001	0.000002	-2.569680
N	0.053717	-2.242419	-0.696607
N	1.915135	1.167731	-0.696603
N	-1.968851	1.074691	-0.696605
C	1.324211	0.461942	-3.004310
C	-0.262053	-1.377765	-3.004312
C	-1.062154	0.915833	-3.004311
C	0.412480	-2.399819	-2.110936
C	1.872067	1.557130	-2.110932
C	-2.284545	0.842695	-2.110934
C	3.038352	0.255643	-0.432647
C	-1.297783	-2.759111	-0.432651
C	-1.740569	2.503469	-0.432646
C	1.010702	-2.979503	0.146447
C	2.074973	2.365045	0.146453
C	-3.085676	0.614457	0.146449
H	2.005360	-0.393793	-3.001078
H	-1.343716	-1.539791	-3.001080
H	-0.661641	1.933593	-3.001076
H	1.291645	0.823227	-4.046195
H	0.067111	-1.530204	-4.046197
H	-1.358753	0.706989	-4.046196
H	1.500196	-2.303987	-2.189393
H	1.245216	2.451204	-2.189388
H	-2.745410	-0.147211	-2.189392
H	0.166331	-3.411030	-2.469852
H	2.870876	1.849565	-2.469846
H	-3.037204	1.561472	-2.469849
H	3.998570	0.743864	-0.637934
H	-1.355080	-3.834794	-0.637940
H	-2.643490	3.090932	-0.637934
H	3.014521	-0.054111	0.610342
H	-1.554121	-2.583597	0.610338
H	-1.460400	2.637705	0.610343
H	2.968665	-0.634817	-1.060491
H	-2.034100	-2.253529	-1.060494
H	-0.934564	2.888349	-1.060489
H	2.015913	-2.588049	-0.004427
H	-3.249273	-0.451808	-0.004427
H	1.233360	3.039857	-0.004422
H	2.094883	2.071432	1.191625
H	0.746471	-2.849941	1.191620
H	-2.841356	0.778505	1.191622
H	1.006622	-4.046653	-0.102065
H	3.001193	2.895087	-0.102057
H	-4.007815	1.151566	-0.102061

Compound **3**, [Mn(me$_6$tren)I], geometry optimization with the largest Positive electric field

Mn	-0.000000	0.000000	0.237855
I	0.000001	-0.000000	-2.426513
N	-0.000001	0.000000	2.574916
N	0.142839	2.230800	0.695474
N	1.860510	-1.239103	0.695475
N	-2.003350	-0.991697	0.695473
C	1.311978	-0.511021	3.012116
C	-0.213434	1.391717	3.012116
C	-1.098548	-0.880697	3.012116
C	0.516669	2.379769	2.112937
C	1.802604	-1.637334	2.112938
C	-2.319276	-0.742434	2.112936
C	3.025392	-0.383581	0.428388
C	-1.180505	2.811857	0.428387
C	-1.844887	-2.428276	0.428386
C	1.128541	2.913318	-0.155891
C	1.958737	-2.434004	-0.155890
C	-3.087279	-0.479314	-0.155892
H	2.022582	0.310888	2.990206
H	-1.280530	1.596164	2.990205
H	-0.742055	-1.907052	2.990205
H	1.273876	-0.858337	4.045212
H	0.106402	1.532379	4.045212
H	-1.380282	-0.674042	4.045212
H	1.593510	2.227636	2.183027
H	1.132433	-2.493838	2.183026
H	-2.725945	0.266203	2.183025
H	0.323954	3.399678	2.458389
H	2.782229	-1.980392	2.458390
H	-3.106186	-1.419285	2.458388
H	3.962084	-0.920279	0.618904
H	-1.184058	3.891405	0.618903
H	-2.778027	-2.971126	0.618902
H	3.007216	-0.067690	-0.617768
H	-1.444985	2.638170	-0.617770
H	-1.562229	-2.570480	-0.617770
H	3.008647	0.505469	1.057971
H	-1.942072	2.352831	1.057969
H	-1.066575	-2.858299	1.057970
H	2.116684	2.473519	-0.014906
H	-3.200472	0.596342	-0.014908
H	1.083789	-3.069862	-0.014907
H	1.992894	-2.132048	-1.204645
H	0.849962	2.791920	-1.204646
H	-2.842854	-0.659872	-1.204647
H	1.182716	3.982603	0.082665
H	2.857678	-3.015564	0.082666
H	-4.040395	-0.967040	0.082664
